# *cyclo*-Tri-μ-oxido-tris­{[(η^5^,η^5^)-1,2-bis­(cyclo­penta­dien­yl)-1,1,2,2-tetra­methyl­disilane]zirconium(IV)}: a trimeric disila-bridged oxidozirconocene

**DOI:** 10.1107/S1600536811007094

**Published:** 2011-03-02

**Authors:** Thomas Arnold, Holger Braunschweig, Katrin Gruss

**Affiliations:** a Institut für Anorganische Chemie, Universität Würzburg, Am Hubland, D-97074 Würzburg, Germany

## Abstract

The title compound, [Zr_3_(C_14_H_20_Si_2_)_3_O_3_], consists of three disila-bridged zirconocene units, which are connected *via* an oxide ligand, forming a nearly planar six-membered ring with a maximum displacement of 0.0191 (8) Å. The compound was isolated as a by-product from a mixture of [(C_5_H_4_SiMe_2_)_2_ZrCl_2_] and Li[AlH_4_] in Et_2_O.

## Related literature

For analogous oxido complexes of zirconocene, see: Chiesi-Villa *et al.* (1979 ▶); Mikhailova *et al.* (1993 ▶). For an analogous oxido complex of hafnocene, see: Atwood *et al.* (1982 ▶).
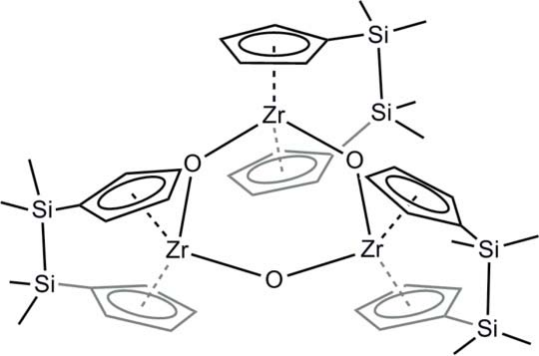


## Experimental

### 

#### Crystal data

[Zr_3_(C_14_H_20_Si_2_)_3_O_3_]
*M* *_r_* = 1055.10
Monoclinic, 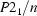

*a* = 8.5399 (7) Å
*b* = 26.667 (2) Å
*c* = 20.9072 (18) Å
β = 95.783 (1)°
*V* = 4737.1 (7) Å^3^
*Z* = 4
Mo *K*α radiation
μ = 0.84 mm^−1^
*T* = 174 K
0.81 × 0.19 × 0.18 mm

#### Data collection

Bruker SMART CCD area-detector diffractometer
Absorption correction: multi-scan (*SADABS*; Bruker, 2001 ▶) *T*_min_ = 0.560, *T*_max_ = 0.860
126251 measured reflections
11796 independent reflections
10301 reflections with *I* > 2σ(*I*)
*R*_int_ = 0.047

#### Refinement

*R*[*F* ^2^ > 2σ(*F* ^2^)] = 0.027
*wR*(*F* ^2^) = 0.069
*S* = 1.02
11796 reflections
499 parameters
18 restraints
H-atom parameters constrained
Δρ_max_ = 0.60 e Å^−3^
Δρ_min_ = −0.30 e Å^−3^

### 

Data collection: *SMART-NT* (Bruker, 2007 ▶); cell refinement: *SAINT-Plus-NT* (Bruker, 2007 ▶); data reduction: *SAINT-Plus-NT*; program(s) used to solve structure: *SHELXS97* (Sheldrick, 2008 ▶); program(s) used to refine structure: *SHELXL97* (Sheldrick, 2008 ▶); molecular graphics: *ORTEP-3* (Farrugia, 1997 ▶); software used to prepare material for publication: *SHELXL97*.

## Supplementary Material

Crystal structure: contains datablocks I, global. DOI: 10.1107/S1600536811007094/su2258sup1.cif

Structure factors: contains datablocks I. DOI: 10.1107/S1600536811007094/su2258Isup2.hkl

Additional supplementary materials:  crystallographic information; 3D view; checkCIF report

## Figures and Tables

**Fig. 1.**
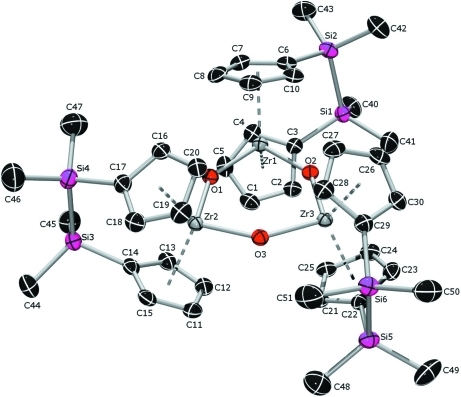
The molecular structure of the title compound with displacement ellipsoids drawn at the 50% probability level. Hydrogen atoms have been omitted for clarity.

## supplementary crystallographic information

### Comment

The zirconium and oxygen atoms of the title compound (Fig. 1) form a nearly planar six-membered ring with a maximal displacement of 0.0191 (8) Å. This is comparable to the same conformation of the analagous oxo-zirconocene structures, reported on by (Chiesi-Villa *et al.*, 1979) [(I), 0.030 Å] and (Mikhailova *et al.*, 1993) [(II), 0.023 Å].

The Zr—O bond lengths range from 1.9650 (13) to 1.9728 (13) Å and the Zr—O—Zr angles [141.73 (7)°, 142.00 (7)° and 142.22 (8)°] are assimilable to bond lengths and angles in the related oxo-zirconocenes (I) and (II).

The dihedral angles between the cyclopentadienyl rings of each zirconocenophane unit are 52.44 (7), 53.23 (8) and 54.58 (7)° and the Zr—Cp distances range from 2.2756 (9) to 2.2997 (9) Å, both close to the values found for the aforementioned compounds.

The structure of an analogous hafnocene oxo-complex has been reported on by (Atwood *et al.*, 1982).

### Experimental

Li[AlH_4_] (50.0 mg, 1.32 mmol, 5.40 eq.) was added to a solution of tetramethyldisilane-l,2-diyl-dicyclopentadienylzirconocendichloride (100 mg, 0.25 mmol) in Et_2_O (5 ml) under stirring at 195 K. The reaction mixture was allowed to warm to room temperature. All volatiles were removed under reduced pressure. The residue was extracted with toluene (3 × 5 ml) and washed with cold hexane (3 × 1 ml). The title compound was obtained as a side product due to oxygen and/or moisture contamination. Colourless crystals suitable for X-ray analysis were grown from a toluene solution a 238 K.

### Refinement

The H atoms were placed at idealized positions and treated as riding atoms: C–H = 0.98 and 1.00 Å for CH_3_ and CH H-atoms, respectively, with *U*_iso_(H) = k × U eq (C), where k = 1.5 for CH_3_ H-atoms and k = 1.2 for all other H-atoms.

### Crystal data

**Table:**

[Zr_3_(C_14_H_20_Si_2_)_3_O_3_]	*F*(000) = 2160
*M**_r_* = 1055.10	*D*_x_ = 1.479 Mg m^−^^3^
Monoclinic, *P*2_1_/*n*	Mo *K*α radiation, λ = 0.71073 Å
Hall symbol: -P 2yn	Cell parameters from 7751 reflections
*a* = 8.5399 (7) Å	θ = 2.5–28.2°
*b* = 26.667 (2) Å	µ = 0.84 mm^−^^1^
*c* = 20.9072 (18) Å	*T* = 174 K
β = 95.783 (1)°	Block, colourless
*V* = 4737.1 (7) Å^3^	0.81 × 0.19 × 0.18 mm
*Z* = 4	

### Data collection

**Table:**

Bruker SMART CCD area-detector diffractometer	11796 independent reflections
Radiation source: sealed tube	10301 reflections with *I* > 2σ(*I*)
graphite	*R*_int_ = 0.047
φ and ω scans	θ_max_ = 28.3°, θ_min_ = 1.5°
Absorption correction: multi-scan (*SADABS*; Bruker, 2001)	*h* = −11→11
*T*_min_ = 0.560, *T*_max_ = 0.860	*k* = −35→35
126251 measured reflections	*l* = −27→27

### Refinement

**Table:**

Refinement on *F*^2^	Primary atom site location: structure-invariant direct methods
Least-squares matrix: full	Secondary atom site location: difference Fourier map
*R*[*F*^2^ > 2σ(*F*^2^)] = 0.027	Hydrogen site location: inferred from neighbouring sites
*wR*(*F*^2^) = 0.069	H-atom parameters constrained
*S* = 1.02	*w* = 1/[σ^2^(*F*_o_^2^) + (0.032*P*)^2^ + 3.3311*P*] where *P* = (*F*_o_^2^ + 2*F*_c_^2^)/3
11796 reflections	(Δ/σ)_max_ = 0.046
499 parameters	Δρ_max_ = 0.60 e Å^−^^3^
18 restraints	Δρ_min_ = −0.30 e Å^−^^3^

### Special details

**Table:**

Geometry. All e.s.d.'s (except the e.s.d. in the dihedral angle between two l.s. planes) are estimated using the full covariance matrix. The cell e.s.d.'s are taken into account individually in the estimation of e.s.d.'s in distances, angles and torsion angles; correlations between e.s.d.'s in cell parameters are only used when they are defined by crystal symmetry. An approximate (isotropic) treatment of cell e.s.d.'s is used for estimating e.s.d.'s involving l.s. planes.
Refinement. Refinement of *F*^2^ against ALL reflections. The weighted *R*-factor *wR* and goodness of fit *S* are based on *F*^2^, conventional *R*-factors *R* are based on *F*, with *F* set to zero for negative *F*^2^. The threshold expression of *F*^2^ > σ(*F*^2^) is used only for calculating *R*-factors(gt) *etc*. and is not relevant to the choice of reflections for refinement. *R*-factors based on *F*^2^ are statistically about twice as large as those based on *F*, and *R*- factors based on ALL data will be even larger.

### Fractional atomic coordinates and isotropic or equivalent isotropic displacement parameters (Å^2^)

**Table:**

	*x*	*y*	*z*	*U*_iso_*/*U*_eq_	
C1	0.7213 (2)	0.22238 (8)	0.51654 (11)	0.0328 (5)	
H1A	0.6603	0.2539	0.5066	0.039*	
C2	0.7067 (2)	0.19067 (8)	0.56930 (10)	0.0284 (4)	
H2A	0.6329	0.1961	0.6029	0.034*	
C3	0.7954 (2)	0.14646 (7)	0.56227 (10)	0.0261 (4)	
C4	0.8624 (2)	0.15173 (8)	0.50292 (10)	0.0299 (4)	
H4A	0.9167	0.1244	0.4810	0.036*	
C5	0.8176 (3)	0.19807 (9)	0.47515 (10)	0.0342 (5)	
H5A	0.8355	0.2092	0.4308	0.041*	
C6	1.1834 (2)	0.14016 (7)	0.61006 (10)	0.0282 (4)	
C7	1.2177 (2)	0.15608 (8)	0.54777 (11)	0.0317 (4)	
H7A	1.2087	0.1345	0.5084	0.038*	
C8	1.2856 (2)	0.20391 (8)	0.55204 (12)	0.0337 (5)	
H8A	1.3357	0.2214	0.5170	0.040*	
C9	1.2957 (2)	0.21886 (8)	0.61711 (11)	0.0315 (4)	
H9A	1.3551	0.2486	0.6359	0.038*	
C10	1.2351 (2)	0.17991 (8)	0.65234 (10)	0.0287 (4)	
H10A	1.2421	0.1782	0.7003	0.034*	
C11	0.7759 (3)	0.41182 (8)	0.54850 (11)	0.0327 (5)	
H11A	0.7300	0.4315	0.5828	0.039*	
C12	0.7283 (2)	0.36354 (9)	0.52729 (11)	0.0320 (5)	
H12A	0.6427	0.3433	0.5441	0.038*	
C13	0.8012 (2)	0.35271 (8)	0.47164 (10)	0.0297 (4)	
H13A	0.7761	0.3233	0.4428	0.036*	
C14	0.8978 (2)	0.39380 (8)	0.45760 (10)	0.0277 (4)	
C15	0.8801 (3)	0.43033 (8)	0.50643 (10)	0.0305 (4)	
H15A	0.9204	0.4655	0.5062	0.037*	
C16	1.3216 (2)	0.34470 (8)	0.55898 (10)	0.0285 (4)	
H16A	1.3731	0.3129	0.5467	0.034*	
C17	1.2791 (2)	0.38501 (8)	0.51635 (10)	0.0284 (4)	
C18	1.2374 (2)	0.42519 (8)	0.55611 (11)	0.0319 (4)	
H18A	1.2150	0.4603	0.5412	0.038*	
C19	1.2514 (2)	0.40956 (8)	0.62041 (11)	0.0324 (4)	
H19A	1.2441	0.4317	0.6586	0.039*	
C20	1.3033 (2)	0.35943 (8)	0.62218 (10)	0.0299 (4)	
H20A	1.3414	0.3403	0.6619	0.036*	
C21	0.6492 (2)	0.33987 (8)	0.68829 (10)	0.0262 (4)	
H21A	0.6292	0.3695	0.6594	0.031*	
C22	0.6743 (2)	0.34198 (8)	0.75636 (10)	0.0263 (4)	
C23	0.6692 (2)	0.29136 (8)	0.77796 (11)	0.0314 (4)	
H23A	0.6692	0.2807	0.8238	0.038*	
C24	0.6437 (2)	0.25944 (8)	0.72465 (11)	0.0315 (4)	
H24A	0.6199	0.2228	0.7261	0.038*	
C25	0.6322 (2)	0.28962 (8)	0.66893 (11)	0.0284 (4)	
H25A	0.5967	0.2779	0.6244	0.034*	
C26	1.1070 (3)	0.25610 (8)	0.80557 (10)	0.0321 (5)	
H26A	1.1140	0.2193	0.8147	0.039*	
C27	1.2057 (2)	0.28335 (8)	0.76768 (11)	0.0320 (5)	
H27A	1.2935	0.2690	0.7451	0.038*	
C28	1.1749 (2)	0.33442 (8)	0.77549 (10)	0.0289 (4)	
H28A	1.2379	0.3624	0.7592	0.035*	
C29	1.0565 (2)	0.34020 (8)	0.81813 (9)	0.0267 (4)	
C30	1.0175 (3)	0.29074 (8)	0.83664 (10)	0.0291 (4)	
H30A	0.9503	0.2824	0.8717	0.035*	
O1	1.02907 (16)	0.28389 (5)	0.54315 (7)	0.0248 (3)	
O3	0.96712 (16)	0.34911 (5)	0.65279 (6)	0.0243 (3)	
O2	0.94862 (15)	0.23813 (5)	0.66530 (6)	0.0243 (3)	
C40	0.7028 (3)	0.03762 (9)	0.58643 (13)	0.0459 (6)	
H40A	0.5911	0.0467	0.5803	0.069*	
H40B	0.7175	0.0096	0.6167	0.069*	
H40C	0.7381	0.0277	0.5451	0.069*	
C50	1.0053 (3)	0.40639 (11)	0.93348 (12)	0.0506 (7)	
H50A	1.1182	0.4099	0.9467	0.076*	
H50B	0.9654	0.3763	0.9532	0.076*	
H50C	0.9495	0.4359	0.9474	0.076*	
C44	0.9865 (3)	0.46377 (9)	0.35259 (12)	0.0438 (6)	
H44A	0.8736	0.4700	0.3426	0.066*	
H44B	1.0389	0.4662	0.3131	0.066*	
H44C	1.0309	0.4888	0.3837	0.066*	
C42	1.1673 (3)	0.06300 (10)	0.71522 (13)	0.0490 (6)	
H42A	1.2826	0.0622	0.7195	0.074*	
H42B	1.1270	0.0300	0.7260	0.074*	
H42C	1.1314	0.0882	0.7446	0.074*	
C41	0.7481 (4)	0.11258 (11)	0.69697 (13)	0.0524 (7)	
H41A	0.6362	0.1212	0.6897	0.079*	
H41B	0.8079	0.1419	0.7139	0.079*	
H41C	0.7623	0.0850	0.7280	0.079*	
C51	1.0762 (3)	0.45204 (10)	0.80427 (15)	0.0524 (7)	
H51A	1.1889	0.4510	0.8189	0.079*	
H51B	1.0329	0.4845	0.8155	0.079*	
H51C	1.0611	0.4475	0.7575	0.079*	
C45	0.9447 (3)	0.35051 (10)	0.32789 (12)	0.0462 (6)	
H45A	0.8315	0.3549	0.3163	0.069*	
H45B	0.9643	0.3172	0.3467	0.069*	
H45C	1.0003	0.3538	0.2893	0.069*	
C43	1.1529 (3)	0.03121 (9)	0.57251 (14)	0.0477 (6)	
H43A	1.2680	0.0294	0.5752	0.072*	
H43B	1.1114	0.0407	0.5288	0.072*	
H43C	1.1106	−0.0016	0.5831	0.072*	
C46	1.4204 (4)	0.43439 (13)	0.40350 (16)	0.0652 (9)	
H46A	1.5272	0.4263	0.4223	0.098*	
H46B	1.3895	0.4672	0.4192	0.098*	
H46C	1.4178	0.4353	0.3565	0.098*	
C47	1.3436 (4)	0.32184 (12)	0.40230 (15)	0.0629 (8)	
H47A	1.4512	0.3153	0.4213	0.094*	
H47B	1.3399	0.3204	0.3553	0.094*	
H47C	1.2725	0.2965	0.4172	0.094*	
C49	0.5711 (3)	0.39843 (13)	0.87020 (13)	0.0566 (8)	
H49A	0.4614	0.3999	0.8512	0.085*	
H49B	0.5930	0.4271	0.8990	0.085*	
H49C	0.5884	0.3672	0.8947	0.085*	
C48	0.6556 (4)	0.45442 (10)	0.74902 (14)	0.0559 (7)	
H48A	0.5470	0.4509	0.7294	0.084*	
H48B	0.7273	0.4547	0.7153	0.084*	
H48C	0.6663	0.4859	0.7733	0.084*	
Si1	0.82032 (7)	0.09271 (2)	0.61928 (3)	0.02879 (12)	
Si2	1.09300 (7)	0.07936 (2)	0.63085 (3)	0.03072 (13)	
Si3	1.01722 (7)	0.39946 (2)	0.38769 (3)	0.02995 (12)	
Si4	1.28066 (8)	0.38554 (3)	0.42733 (3)	0.03512 (14)	
Si5	0.70555 (7)	0.40033 (2)	0.80469 (3)	0.03030 (12)	
Si6	0.97278 (7)	0.40082 (2)	0.84410 (3)	0.02893 (12)	
Zr1	1.00124 (2)	0.216851 (7)	0.579718 (9)	0.02146 (5)	
Zr2	1.02592 (2)	0.355885 (7)	0.564699 (9)	0.02160 (5)	
Zr3	0.91738 (2)	0.298490 (7)	0.716405 (9)	0.02088 (5)	

### Atomic displacement parameters (Å^2^)

**Table:**

	*U*^11^	*U*^22^	*U*^33^	*U*^12^	*U*^13^	*U*^23^
C1	0.0231 (10)	0.0306 (10)	0.0422 (12)	−0.0019 (8)	−0.0082 (9)	0.0036 (9)
C2	0.0185 (9)	0.0303 (10)	0.0356 (11)	−0.0030 (8)	−0.0016 (8)	−0.0022 (8)
C3	0.0228 (9)	0.0280 (10)	0.0271 (10)	−0.0041 (7)	−0.0001 (7)	−0.0026 (8)
C4	0.0290 (10)	0.0337 (11)	0.0266 (10)	−0.0062 (8)	0.0006 (8)	−0.0069 (8)
C5	0.0322 (11)	0.0414 (12)	0.0273 (10)	−0.0116 (9)	−0.0048 (8)	0.0020 (9)
C6	0.0229 (10)	0.0264 (10)	0.0341 (11)	0.0051 (7)	−0.0021 (8)	−0.0025 (8)
C7	0.0249 (10)	0.0341 (11)	0.0364 (11)	0.0037 (8)	0.0045 (8)	−0.0058 (9)
C8	0.0204 (10)	0.0373 (12)	0.0448 (13)	0.0011 (8)	0.0096 (9)	0.0005 (9)
C9	0.0167 (9)	0.0308 (10)	0.0459 (12)	−0.0003 (8)	−0.0025 (8)	−0.0027 (9)
C10	0.0228 (10)	0.0270 (10)	0.0348 (11)	0.0043 (8)	−0.0047 (8)	−0.0020 (8)
C11	0.0273 (10)	0.0371 (11)	0.0334 (11)	0.0110 (9)	0.0016 (8)	0.0044 (9)
C12	0.0178 (9)	0.0413 (12)	0.0360 (11)	0.0016 (8)	−0.0013 (8)	0.0094 (9)
C13	0.0239 (10)	0.0330 (11)	0.0305 (10)	−0.0016 (8)	−0.0051 (8)	0.0044 (8)
C14	0.0267 (10)	0.0291 (10)	0.0263 (10)	0.0029 (8)	−0.0020 (8)	0.0060 (8)
C15	0.0315 (11)	0.0268 (10)	0.0324 (11)	0.0066 (8)	−0.0008 (8)	0.0074 (8)
C16	0.0163 (9)	0.0318 (10)	0.0373 (11)	−0.0007 (7)	0.0022 (8)	0.0048 (8)
C17	0.0215 (9)	0.0305 (10)	0.0334 (10)	−0.0045 (8)	0.0035 (8)	0.0037 (8)
C18	0.0268 (10)	0.0260 (10)	0.0424 (12)	−0.0056 (8)	0.0017 (9)	0.0022 (9)
C19	0.0255 (10)	0.0355 (11)	0.0359 (11)	−0.0084 (8)	0.0005 (8)	−0.0049 (9)
C20	0.0163 (9)	0.0402 (12)	0.0321 (10)	−0.0026 (8)	−0.0030 (7)	0.0067 (9)
C21	0.0180 (9)	0.0300 (10)	0.0299 (10)	0.0026 (7)	−0.0015 (7)	−0.0008 (8)
C22	0.0199 (9)	0.0304 (10)	0.0288 (10)	0.0018 (7)	0.0041 (7)	−0.0004 (8)
C23	0.0235 (10)	0.0383 (12)	0.0334 (11)	0.0000 (8)	0.0079 (8)	0.0039 (9)
C24	0.0205 (9)	0.0290 (10)	0.0456 (12)	−0.0036 (8)	0.0056 (8)	−0.0004 (9)
C25	0.0163 (9)	0.0323 (10)	0.0358 (11)	0.0000 (7)	−0.0015 (8)	−0.0054 (8)
C26	0.0320 (11)	0.0303 (11)	0.0317 (11)	0.0055 (8)	−0.0085 (8)	0.0012 (8)
C27	0.0204 (10)	0.0420 (12)	0.0322 (11)	0.0054 (8)	−0.0043 (8)	−0.0045 (9)
C28	0.0217 (9)	0.0355 (11)	0.0282 (10)	−0.0028 (8)	−0.0038 (8)	−0.0027 (8)
C29	0.0249 (10)	0.0301 (10)	0.0241 (9)	0.0000 (8)	−0.0023 (7)	−0.0009 (8)
C30	0.0306 (11)	0.0328 (11)	0.0224 (9)	0.0018 (8)	−0.0043 (8)	0.0037 (8)
O1	0.0232 (7)	0.0230 (7)	0.0280 (7)	−0.0003 (5)	0.0016 (5)	0.0009 (5)
O3	0.0220 (7)	0.0251 (7)	0.0256 (7)	0.0002 (5)	0.0010 (5)	0.0012 (5)
O2	0.0219 (7)	0.0249 (7)	0.0258 (7)	0.0005 (5)	0.0014 (5)	−0.0006 (5)
C40	0.0489 (15)	0.0333 (12)	0.0532 (15)	−0.0121 (11)	−0.0068 (12)	0.0075 (11)
C50	0.0558 (16)	0.0606 (17)	0.0325 (12)	0.0214 (13)	−0.0092 (11)	−0.0134 (11)
C44	0.0572 (16)	0.0370 (12)	0.0362 (12)	−0.0021 (11)	0.0006 (11)	0.0092 (10)
C42	0.0546 (16)	0.0438 (14)	0.0457 (14)	0.0000 (12)	−0.0094 (12)	0.0085 (11)
C41	0.0704 (19)	0.0514 (16)	0.0383 (14)	0.0098 (14)	0.0194 (13)	0.0034 (12)
C51	0.0473 (15)	0.0325 (13)	0.079 (2)	−0.0049 (11)	0.0134 (14)	0.0017 (13)
C45	0.0543 (16)	0.0462 (14)	0.0378 (13)	−0.0060 (12)	0.0033 (11)	−0.0084 (11)
C43	0.0515 (16)	0.0320 (12)	0.0596 (16)	0.0025 (11)	0.0054 (13)	−0.0100 (11)
C46	0.0470 (16)	0.088 (2)	0.0612 (17)	−0.0172 (15)	0.0080 (13)	0.0302 (16)
C47	0.0634 (18)	0.0707 (19)	0.0531 (16)	0.0266 (15)	−0.0004 (14)	−0.0145 (14)
C49	0.0445 (15)	0.082 (2)	0.0451 (15)	0.0015 (14)	0.0133 (12)	−0.0245 (14)
C48	0.0704 (18)	0.0362 (13)	0.0553 (16)	0.0094 (12)	−0.0220 (14)	−0.0030 (12)
Si1	0.0312 (3)	0.0253 (3)	0.0298 (3)	−0.0025 (2)	0.0026 (2)	0.0005 (2)
Si2	0.0323 (3)	0.0238 (3)	0.0349 (3)	0.0012 (2)	−0.0026 (2)	−0.0009 (2)
Si3	0.0334 (3)	0.0306 (3)	0.0255 (3)	−0.0012 (2)	0.0018 (2)	0.0033 (2)
Si4	0.0310 (3)	0.0436 (4)	0.0316 (3)	0.0013 (3)	0.0072 (2)	0.0056 (3)
Si5	0.0303 (3)	0.0314 (3)	0.0286 (3)	0.0061 (2)	−0.0003 (2)	−0.0051 (2)
Si6	0.0300 (3)	0.0277 (3)	0.0283 (3)	0.0008 (2)	−0.0015 (2)	−0.0035 (2)
Zr1	0.01822 (9)	0.02122 (9)	0.02454 (9)	−0.00059 (6)	0.00019 (7)	−0.00107 (7)
Zr2	0.01809 (9)	0.02180 (9)	0.02452 (9)	−0.00035 (6)	0.00018 (7)	0.00312 (7)
Zr3	0.01773 (9)	0.02240 (9)	0.02217 (9)	0.00036 (6)	0.00037 (6)	0.00030 (7)

### Geometric parameters (Å, °)

**Table:**

C1—C2	1.405 (3)	C25—H25A	1.0000
C1—C5	1.410 (3)	C26—C30	1.400 (3)
C1—Zr1	2.616 (2)	C26—C27	1.414 (3)
C1—H1A	1.0000	C26—Zr3	2.602 (2)
C2—C3	1.417 (3)	C26—H26A	1.0000
C2—Zr1	2.5982 (19)	C27—C28	1.400 (3)
C2—H2A	1.0000	C27—Zr3	2.616 (2)
C3—C4	1.425 (3)	C27—H27A	1.0000
C3—Si1	1.862 (2)	C28—C29	1.422 (3)
C3—Zr1	2.5724 (19)	C28—Zr3	2.5946 (19)
C4—C5	1.402 (3)	C28—H28A	1.0000
C4—Zr1	2.572 (2)	C29—C30	1.423 (3)
C4—H4A	1.0000	C29—Si6	1.870 (2)
C5—Zr1	2.608 (2)	C29—Zr3	2.5812 (19)
C5—H5A	1.0000	C30—Zr3	2.581 (2)
C6—C10	1.422 (3)	C30—H30A	1.0000
C6—C7	1.428 (3)	O1—Zr1	1.9681 (13)
C6—Si2	1.866 (2)	O1—Zr2	1.9728 (13)
C6—Zr1	2.609 (2)	O3—Zr2	1.9654 (14)
C7—C8	1.401 (3)	O3—Zr3	1.9711 (13)
C7—Zr1	2.596 (2)	O2—Zr3	1.9650 (13)
C7—H7A	1.0000	O2—Zr1	1.9716 (13)
C8—C9	1.412 (3)	C40—Si1	1.871 (2)
C8—Zr1	2.576 (2)	C40—H40A	0.9800
C8—H8A	1.0000	C40—H40B	0.9800
C9—C10	1.402 (3)	C40—H40C	0.9800
C9—Zr1	2.558 (2)	C50—Si6	1.867 (2)
C9—H9A	1.0000	C50—H50A	0.9800
C10—Zr1	2.579 (2)	C50—H50B	0.9800
C10—H10A	1.0000	C50—H50C	0.9800
C11—C15	1.402 (3)	C44—Si3	1.874 (2)
C11—C12	1.408 (3)	C44—H44A	0.9800
C11—Zr2	2.599 (2)	C44—H44B	0.9800
C11—H11A	1.0000	C44—H44C	0.9800
C12—C13	1.404 (3)	C42—Si2	1.865 (3)
C12—Zr2	2.591 (2)	C42—H42A	0.9800
C12—H12A	1.0000	C42—H42B	0.9800
C13—C14	1.420 (3)	C42—H42C	0.9800
C13—Zr2	2.593 (2)	C41—Si1	1.871 (3)
C13—H13A	1.0000	C41—H41A	0.9800
C14—C15	1.430 (3)	C41—H41B	0.9800
C14—Si3	1.870 (2)	C41—H41C	0.9800
C14—Zr2	2.5960 (19)	C51—Si6	1.867 (3)
C15—Zr2	2.5827 (19)	C51—H51A	0.9800
C15—H15A	1.0000	C51—H51B	0.9800
C16—C20	1.402 (3)	C51—H51C	0.9800
C16—C17	1.420 (3)	C45—Si3	1.869 (2)
C16—Zr2	2.5578 (19)	C45—H45A	0.9800
C16—H16A	1.0000	C45—H45B	0.9800
C17—C18	1.423 (3)	C45—H45C	0.9800
C17—Si4	1.863 (2)	C43—Si2	1.877 (2)
C17—Zr2	2.597 (2)	C43—H43A	0.9800
C18—C19	1.401 (3)	C43—H43B	0.9800
C18—Zr2	2.603 (2)	C43—H43C	0.9800
C18—H18A	1.0000	C46—Si4	1.868 (3)
C19—C20	1.408 (3)	C46—H46A	0.9800
C19—Zr2	2.581 (2)	C46—H46B	0.9800
C19—H19A	1.0000	C46—H46C	0.9800
C20—Zr2	2.5481 (19)	C47—Si4	1.872 (3)
C20—H20A	1.0000	C47—H47A	0.9800
C21—C25	1.403 (3)	C47—H47B	0.9800
C21—C22	1.419 (3)	C47—H47C	0.9800
C21—Zr3	2.5571 (19)	C49—Si5	1.875 (3)
C21—H21A	1.0000	C49—H49A	0.9800
C22—C23	1.426 (3)	C49—H49B	0.9800
C22—Si5	1.860 (2)	C49—H49C	0.9800
C22—Zr3	2.5890 (19)	C48—Si5	1.876 (3)
C23—C24	1.402 (3)	C48—H48A	0.9800
C23—Zr3	2.595 (2)	C48—H48B	0.9800
C23—H23A	1.0000	C48—H48C	0.9800
C24—C25	1.411 (3)	Si1—Si2	2.3440 (9)
C24—Zr3	2.580 (2)	Si3—Si4	2.3474 (9)
C24—H24A	1.0000	Si5—Si6	2.3469 (9)
C25—Zr3	2.547 (2)		
			
C2—C1—C5	107.96 (19)	H49A—C49—H49B	109.5
C2—C1—Zr1	73.67 (12)	Si5—C49—H49C	109.5
C5—C1—Zr1	74.01 (12)	H49A—C49—H49C	109.5
C2—C1—H1A	125.7	H49B—C49—H49C	109.5
C5—C1—H1A	125.7	Si5—C48—H48A	109.5
Zr1—C1—H1A	125.7	Si5—C48—H48B	109.5
C1—C2—C3	109.18 (19)	H48A—C48—H48B	109.5
C1—C2—Zr1	75.06 (12)	Si5—C48—H48C	109.5
C3—C2—Zr1	73.09 (11)	H48A—C48—H48C	109.5
C1—C2—H2A	125.1	H48B—C48—H48C	109.5
C3—C2—H2A	125.1	C3—Si1—C40	110.30 (10)
Zr1—C2—H2A	125.1	C3—Si1—C41	108.13 (11)
C2—C3—C4	105.90 (18)	C40—Si1—C41	109.23 (14)
C2—C3—Si1	126.99 (16)	C3—Si1—Si2	103.36 (7)
C4—C3—Si1	127.11 (16)	C40—Si1—Si2	114.17 (9)
C2—C3—Zr1	75.10 (11)	C41—Si1—Si2	111.37 (10)
C4—C3—Zr1	73.92 (11)	C42—Si2—C6	108.31 (11)
Si1—C3—Zr1	116.01 (9)	C42—Si2—C43	111.43 (13)
C5—C4—C3	109.33 (19)	C6—Si2—C43	107.26 (11)
C5—C4—Zr1	75.69 (12)	C42—Si2—Si1	111.89 (10)
C3—C4—Zr1	73.93 (11)	C6—Si2—Si1	105.90 (7)
C5—C4—H4A	124.9	C43—Si2—Si1	111.71 (9)
C3—C4—H4A	124.9	C45—Si3—C14	107.25 (11)
Zr1—C4—H4A	124.9	C45—Si3—C44	110.69 (12)
C4—C5—C1	107.62 (19)	C14—Si3—C44	108.30 (11)
C4—C5—Zr1	72.91 (12)	C45—Si3—Si4	111.52 (10)
C1—C5—Zr1	74.67 (12)	C14—Si3—Si4	106.85 (7)
C4—C5—H5A	125.8	C44—Si3—Si4	111.99 (9)
C1—C5—H5A	125.8	C17—Si4—C46	109.92 (13)
Zr1—C5—H5A	125.8	C17—Si4—C47	107.67 (12)
C10—C6—C7	105.43 (19)	C46—Si4—C47	110.44 (17)
C10—C6—Si2	127.80 (17)	C17—Si4—Si3	104.54 (7)
C7—C6—Si2	126.76 (16)	C46—Si4—Si3	114.26 (11)
C10—C6—Zr1	72.96 (11)	C47—Si4—Si3	109.67 (11)
C7—C6—Zr1	73.58 (12)	C22—Si5—C49	108.01 (12)
Si2—C6—Zr1	119.17 (9)	C22—Si5—C48	107.15 (11)
C8—C7—C6	109.53 (19)	C49—Si5—C48	110.88 (15)
C8—C7—Zr1	73.51 (12)	C22—Si5—Si6	106.07 (7)
C6—C7—Zr1	74.57 (11)	C49—Si5—Si6	112.91 (10)
C8—C7—H7A	125.0	C48—Si5—Si6	111.47 (11)
C6—C7—H7A	125.0	C50—Si6—C51	111.02 (15)
Zr1—C7—H7A	125.0	C50—Si6—C29	109.67 (11)
C7—C8—C9	107.6 (2)	C51—Si6—C29	106.92 (11)
C7—C8—Zr1	75.07 (12)	C50—Si6—Si5	113.16 (10)
C9—C8—Zr1	73.33 (12)	C51—Si6—Si5	109.53 (10)
C7—C8—H8A	125.7	C29—Si6—Si5	106.25 (7)
C9—C8—H8A	125.7	O1—Zr1—O2	97.96 (6)
Zr1—C8—H8A	125.7	O1—Zr1—C9	86.92 (6)
C10—C9—C8	107.89 (19)	O2—Zr1—C9	91.35 (7)
C10—C9—Zr1	75.01 (12)	O1—Zr1—C4	115.91 (6)
C8—C9—Zr1	74.75 (12)	O2—Zr1—C4	128.89 (6)
C10—C9—H9A	125.5	C9—Zr1—C4	125.60 (7)
C8—C9—H9A	125.5	O1—Zr1—C3	135.67 (6)
Zr1—C9—H9A	125.5	O2—Zr1—C3	97.11 (6)
C9—C10—C6	109.5 (2)	C9—Zr1—C3	134.05 (7)
C9—C10—Zr1	73.32 (12)	C4—Zr1—C3	32.15 (6)
C6—C10—Zr1	75.24 (11)	O1—Zr1—C8	83.16 (6)
C9—C10—H10A	125.0	O2—Zr1—C8	123.28 (7)
C6—C10—H10A	125.0	C9—Zr1—C8	31.93 (7)
Zr1—C10—H10A	125.0	C4—Zr1—C8	98.92 (7)
C15—C11—C12	107.8 (2)	C3—Zr1—C8	121.11 (7)
C15—C11—Zr2	73.67 (12)	O1—Zr1—C10	117.32 (6)
C12—C11—Zr2	73.96 (12)	O2—Zr1—C10	79.01 (6)
C15—C11—H11A	125.7	C9—Zr1—C10	31.68 (7)
C12—C11—H11A	125.7	C4—Zr1—C10	112.98 (7)
Zr2—C11—H11A	125.7	C3—Zr1—C10	106.39 (6)
C13—C12—C11	108.11 (19)	C8—Zr1—C10	52.38 (7)
C13—C12—Zr2	74.36 (12)	O1—Zr1—C7	110.50 (6)
C11—C12—Zr2	74.55 (12)	O2—Zr1—C7	130.28 (6)
C13—C12—H12A	125.5	C9—Zr1—C7	52.27 (7)
C11—C12—H12A	125.5	C4—Zr1—C7	73.34 (7)
Zr2—C12—H12A	125.5	C3—Zr1—C7	90.11 (7)
C12—C13—C14	109.16 (19)	C8—Zr1—C7	31.42 (7)
C12—C13—Zr2	74.21 (12)	C10—Zr1—C7	51.97 (7)
C14—C13—Zr2	74.23 (11)	O1—Zr1—C2	111.43 (6)
C12—C13—H13A	125.1	O2—Zr1—C2	81.15 (6)
C14—C13—H13A	125.1	C9—Zr1—C2	160.89 (7)
Zr2—C13—H13A	125.1	C4—Zr1—C2	52.03 (7)
C13—C14—C15	105.79 (18)	C3—Zr1—C2	31.81 (6)
C13—C14—Si3	126.71 (16)	C8—Zr1—C2	150.67 (7)
C15—C14—Si3	127.46 (16)	C10—Zr1—C2	129.21 (7)
C13—C14—Zr2	74.00 (11)	C7—Zr1—C2	121.27 (7)
C15—C14—Zr2	73.46 (11)	O1—Zr1—C5	86.26 (6)
Si3—C14—Zr2	119.34 (9)	O2—Zr1—C5	130.04 (7)
C11—C15—C14	109.12 (19)	C9—Zr1—C5	138.60 (8)
C11—C15—Zr2	74.93 (12)	C4—Zr1—C5	31.40 (7)
C14—C15—Zr2	74.48 (11)	C3—Zr1—C5	52.86 (7)
C11—C15—H15A	125.0	C8—Zr1—C5	106.68 (8)
C14—C15—H15A	125.0	C10—Zr1—C5	141.70 (7)
Zr2—C15—H15A	125.0	C7—Zr1—C5	92.75 (7)
C20—C16—C17	109.48 (19)	C2—Zr1—C5	51.88 (7)
C20—C16—Zr2	73.68 (11)	O1—Zr1—C6	135.58 (6)
C17—C16—Zr2	75.52 (11)	O2—Zr1—C6	100.94 (6)
C20—C16—H16A	124.9	C9—Zr1—C6	53.01 (7)
C17—C16—H16A	124.9	C4—Zr1—C6	81.29 (7)
Zr2—C16—H16A	124.9	C3—Zr1—C6	81.06 (7)
C16—C17—C18	105.46 (19)	C8—Zr1—C6	52.92 (7)
C16—C17—Si4	127.28 (17)	C10—Zr1—C6	31.80 (6)
C18—C17—Si4	127.26 (16)	C7—Zr1—C6	31.85 (7)
C16—C17—Zr2	72.51 (11)	C2—Zr1—C6	110.99 (7)
C18—C17—Zr2	74.37 (12)	C5—Zr1—C6	110.29 (7)
Si4—C17—Zr2	118.76 (9)	O1—Zr1—C1	83.84 (6)
C19—C18—C17	109.53 (19)	O2—Zr1—C1	99.31 (7)
C19—C18—Zr2	73.44 (12)	C9—Zr1—C1	166.74 (7)
C17—C18—Zr2	73.86 (11)	C4—Zr1—C1	51.87 (7)
C19—C18—H18A	125.0	C3—Zr1—C1	52.63 (6)
C17—C18—H18A	125.0	C8—Zr1—C1	136.74 (8)
Zr2—C18—H18A	125.0	C10—Zr1—C1	158.84 (7)
C18—C19—C20	107.7 (2)	C7—Zr1—C1	122.91 (7)
C18—C19—Zr2	75.20 (12)	C2—Zr1—C1	31.27 (7)
C20—C19—Zr2	72.79 (12)	C5—Zr1—C1	31.32 (7)
C18—C19—H19A	125.8	C6—Zr1—C1	131.23 (7)
C20—C19—H19A	125.8	O3—Zr2—O1	97.76 (6)
Zr2—C19—H19A	125.8	O3—Zr2—C20	82.80 (6)
C16—C20—C19	107.82 (19)	O1—Zr2—C20	96.34 (6)
C16—C20—Zr2	74.44 (11)	O3—Zr2—C16	112.32 (6)
C19—C20—Zr2	75.36 (12)	O1—Zr2—C16	80.77 (6)
C16—C20—H20A	125.5	C20—Zr2—C16	31.88 (7)
C19—C20—H20A	125.5	O3—Zr2—C19	82.96 (6)
Zr2—C20—H20A	125.5	O1—Zr2—C19	128.02 (7)
C25—C21—C22	109.14 (18)	C20—Zr2—C19	31.85 (7)
C25—C21—Zr3	73.63 (11)	C16—Zr2—C19	52.45 (7)
C22—C21—Zr3	75.24 (11)	O3—Zr2—C15	110.86 (6)
C25—C21—H21A	125.1	O1—Zr2—C15	131.10 (6)
C22—C21—H21A	125.1	C20—Zr2—C15	125.11 (7)
Zr3—C21—H21A	125.1	C16—Zr2—C15	119.93 (7)
C21—C22—C23	105.90 (18)	C19—Zr2—C15	95.10 (7)
C21—C22—Si5	125.24 (15)	O3—Zr2—C12	87.40 (6)
C23—C22—Si5	128.86 (16)	O1—Zr2—C12	92.48 (7)
C21—C22—Zr3	72.76 (11)	C20—Zr2—C12	167.60 (7)
C23—C22—Zr3	74.28 (11)	C16—Zr2—C12	159.76 (7)
Si5—C22—Zr3	118.30 (9)	C19—Zr2—C12	139.21 (7)
C24—C23—C22	109.29 (19)	C15—Zr2—C12	52.07 (7)
C24—C23—Zr3	73.68 (12)	O3—Zr2—C13	117.41 (6)
C22—C23—Zr3	73.80 (11)	O1—Zr2—C13	79.81 (6)
C24—C23—H23A	125.1	C20—Zr2—C13	159.70 (7)
C22—C23—H23A	125.1	C16—Zr2—C13	128.38 (7)
Zr3—C23—H23A	125.1	C19—Zr2—C13	144.98 (7)
C23—C24—C25	107.60 (19)	C15—Zr2—C13	52.11 (7)
C23—C24—Zr3	74.89 (12)	C12—Zr2—C13	31.42 (7)
C25—C24—Zr3	72.72 (11)	O3—Zr2—C14	135.96 (6)
C23—C24—H24A	125.8	O1—Zr2—C14	101.32 (6)
C25—C24—H24A	125.8	C20—Zr2—C14	133.10 (7)
Zr3—C24—H24A	125.8	C16—Zr2—C14	109.71 (7)
C21—C25—C24	108.06 (19)	C19—Zr2—C14	113.81 (7)
C21—C25—Zr3	74.46 (11)	C15—Zr2—C14	32.06 (7)
C24—C25—Zr3	75.33 (12)	C12—Zr2—C14	52.68 (7)
C21—C25—H25A	125.4	C13—Zr2—C14	31.77 (6)
C24—C25—H25A	125.4	O3—Zr2—C17	133.93 (6)
Zr3—C25—H25A	125.4	O1—Zr2—C17	99.86 (6)
C30—C26—C27	107.76 (19)	C20—Zr2—C17	53.22 (7)
C30—C26—Zr3	73.50 (11)	C16—Zr2—C17	31.97 (6)
C27—C26—Zr3	74.84 (12)	C19—Zr2—C17	52.92 (7)
C30—C26—H26A	125.7	C15—Zr2—C17	88.13 (7)
C27—C26—H26A	125.7	C12—Zr2—C17	133.56 (7)
Zr3—C26—H26A	125.7	C13—Zr2—C17	107.47 (7)
C28—C27—C26	107.74 (19)	C14—Zr2—C17	80.95 (7)
C28—C27—Zr3	73.57 (11)	O3—Zr2—C11	83.73 (6)
C26—C27—Zr3	73.70 (12)	O1—Zr2—C11	123.97 (7)
C28—C27—H27A	125.8	C20—Zr2—C11	138.84 (7)
C26—C27—H27A	125.8	C16—Zr2—C11	149.66 (7)
Zr3—C27—H27A	125.8	C19—Zr2—C11	107.84 (7)
C27—C28—C29	109.43 (19)	C15—Zr2—C11	31.41 (7)
C27—C28—Zr3	75.27 (12)	C12—Zr2—C11	31.49 (7)
C29—C28—Zr3	73.53 (11)	C13—Zr2—C11	52.02 (7)
C27—C28—H28A	125.0	C14—Zr2—C11	52.76 (7)
C29—C28—H28A	125.0	C17—Zr2—C11	118.89 (7)
Zr3—C28—H28A	125.0	O3—Zr2—C18	111.99 (6)
C28—C29—C30	105.70 (18)	O1—Zr2—C18	130.52 (6)
C28—C29—Si6	126.32 (16)	C20—Zr2—C18	52.23 (7)
C30—C29—Si6	127.98 (16)	C16—Zr2—C18	52.00 (7)
C28—C29—Zr3	74.57 (11)	C19—Zr2—C18	31.36 (7)
C30—C29—Zr3	73.97 (11)	C15—Zr2—C18	74.14 (7)
Si6—C29—Zr3	117.01 (9)	C12—Zr2—C18	126.13 (7)
C26—C30—C29	109.36 (19)	C13—Zr2—C18	115.96 (7)
C26—C30—Zr3	75.14 (12)	C14—Zr2—C18	84.20 (7)
C29—C30—Zr3	74.01 (11)	C17—Zr2—C18	31.77 (7)
C26—C30—H30A	125.0	C11—Zr2—C18	98.64 (7)
C29—C30—H30A	125.0	O2—Zr3—O3	98.26 (6)
Zr3—C30—H30A	125.0	O2—Zr3—C25	83.62 (6)
Zr1—O1—Zr2	142.22 (8)	O3—Zr3—C25	93.63 (6)
Zr2—O3—Zr3	142.00 (7)	O2—Zr3—C21	113.33 (6)
Zr3—O2—Zr1	141.73 (7)	O3—Zr3—C21	78.36 (6)
Si1—C40—H40A	109.5	C25—Zr3—C21	31.91 (6)
Si1—C40—H40B	109.5	O2—Zr3—C24	83.06 (6)
H40A—C40—H40B	109.5	O3—Zr3—C24	125.43 (6)
Si1—C40—H40C	109.5	C25—Zr3—C24	31.95 (7)
H40A—C40—H40C	109.5	C21—Zr3—C24	52.64 (7)
H40B—C40—H40C	109.5	O2—Zr3—C30	114.48 (6)
Si6—C50—H50A	109.5	O3—Zr3—C30	129.74 (6)
Si6—C50—H50B	109.5	C25—Zr3—C30	125.60 (7)
H50A—C50—H50B	109.5	C21—Zr3—C30	117.36 (7)
Si6—C50—H50C	109.5	C24—Zr3—C30	96.66 (7)
H50A—C50—H50C	109.5	O2—Zr3—C29	136.44 (6)
H50B—C50—H50C	109.5	O3—Zr3—C29	98.53 (6)
Si3—C44—H44A	109.5	C25—Zr3—C29	134.73 (7)
Si3—C44—H44B	109.5	C21—Zr3—C29	109.31 (6)
H44A—C44—H44B	109.5	C24—Zr3—C29	117.62 (7)
Si3—C44—H44C	109.5	C30—Zr3—C29	32.01 (6)
H44A—C44—H44C	109.5	O2—Zr3—C22	134.40 (6)
H44B—C44—H44C	109.5	O3—Zr3—C22	98.36 (6)
Si2—C42—H42A	109.5	C25—Zr3—C22	53.19 (6)
Si2—C42—H42B	109.5	C21—Zr3—C22	32.00 (6)
H42A—C42—H42B	109.5	C24—Zr3—C22	52.99 (7)
Si2—C42—H42C	109.5	C30—Zr3—C22	85.37 (7)
H42A—C42—H42C	109.5	C29—Zr3—C22	81.87 (6)
H42B—C42—H42C	109.5	O2—Zr3—C28	113.98 (6)
Si1—C41—H41A	109.5	O3—Zr3—C28	80.70 (6)
Si1—C41—H41B	109.5	C25—Zr3—C28	162.02 (7)
H41A—C41—H41B	109.5	C21—Zr3—C28	130.29 (7)
Si1—C41—H41C	109.5	C24—Zr3—C28	147.90 (7)
H41A—C41—H41C	109.5	C30—Zr3—C28	51.98 (7)
H41B—C41—H41C	109.5	C29—Zr3—C28	31.90 (6)
Si6—C51—H51A	109.5	C22—Zr3—C28	110.47 (7)
Si6—C51—H51B	109.5	O2—Zr3—C23	111.84 (6)
H51A—C51—H51B	109.5	O3—Zr3—C23	128.84 (6)
Si6—C51—H51C	109.5	C25—Zr3—C23	52.38 (7)
H51A—C51—H51C	109.5	C21—Zr3—C23	52.28 (7)
H51B—C51—H51C	109.5	C24—Zr3—C23	31.43 (7)
Si3—C45—H45A	109.5	C30—Zr3—C23	73.62 (7)
Si3—C45—H45B	109.5	C29—Zr3—C23	87.36 (7)
H45A—C45—H45B	109.5	C22—Zr3—C23	31.92 (6)
Si3—C45—H45C	109.5	C28—Zr3—C23	119.10 (7)
H45A—C45—H45C	109.5	O2—Zr3—C26	85.69 (6)
H45B—C45—H45C	109.5	O3—Zr3—C26	128.33 (7)
Si2—C43—H43A	109.5	C25—Zr3—C26	137.80 (7)
Si2—C43—H43B	109.5	C21—Zr3—C26	146.19 (7)
H43A—C43—H43B	109.5	C24—Zr3—C26	106.23 (7)
Si2—C43—H43C	109.5	C30—Zr3—C26	31.35 (7)
H43A—C43—H43C	109.5	C29—Zr3—C26	52.79 (7)
H43B—C43—H43C	109.5	C22—Zr3—C26	115.16 (7)
Si4—C46—H46A	109.5	C28—Zr3—C26	51.89 (7)
Si4—C46—H46B	109.5	C23—Zr3—C26	95.36 (7)
H46A—C46—H46B	109.5	O2—Zr3—C27	85.56 (6)
Si4—C46—H46C	109.5	O3—Zr3—C27	97.14 (7)
H46A—C46—H46C	109.5	C25—Zr3—C27	165.72 (7)
H46B—C46—H46C	109.5	C21—Zr3—C27	160.94 (7)
Si4—C47—H47A	109.5	C24—Zr3—C27	137.04 (7)
Si4—C47—H47B	109.5	C30—Zr3—C27	51.88 (7)
H47A—C47—H47B	109.5	C29—Zr3—C27	52.62 (7)
Si4—C47—H47C	109.5	C22—Zr3—C27	133.57 (7)
H47A—C47—H47C	109.5	C28—Zr3—C27	31.17 (7)
H47B—C47—H47C	109.5	C23—Zr3—C27	124.58 (7)
Si5—C49—H49A	109.5	C26—Zr3—C27	31.45 (7)
Si5—C49—H49B	109.5		
			
C5—C1—C2—C3	0.9 (2)	C19—C20—Zr2—O1	174.69 (13)
Zr1—C1—C2—C3	−65.70 (14)	C19—C20—Zr2—C16	113.65 (18)
C5—C1—C2—Zr1	66.62 (15)	C16—C20—Zr2—C19	−113.65 (18)
C1—C2—C3—C4	−1.1 (2)	C16—C20—Zr2—C15	−91.41 (14)
Zr1—C2—C3—C4	−68.05 (13)	C19—C20—Zr2—C15	22.24 (16)
C1—C2—C3—Si1	178.56 (15)	C16—C20—Zr2—C12	−163.9 (3)
Zr1—C2—C3—Si1	111.57 (15)	C19—C20—Zr2—C12	−50.2 (4)
C1—C2—C3—Zr1	66.99 (14)	C16—C20—Zr2—C13	−16.7 (3)
C2—C3—C4—C5	0.8 (2)	C19—C20—Zr2—C13	97.0 (2)
Si1—C3—C4—C5	−178.80 (15)	C16—C20—Zr2—C14	−50.95 (16)
Zr1—C3—C4—C5	−68.06 (15)	C19—C20—Zr2—C14	62.70 (16)
C2—C3—C4—Zr1	68.88 (13)	C16—C20—Zr2—C17	−36.55 (12)
Si1—C3—C4—Zr1	−110.74 (16)	C19—C20—Zr2—C17	77.10 (14)
C3—C4—C5—C1	−0.3 (2)	C16—C20—Zr2—C11	−130.06 (13)
Zr1—C4—C5—C1	−67.18 (15)	C19—C20—Zr2—C11	−16.41 (18)
C3—C4—C5—Zr1	66.91 (14)	C16—C20—Zr2—C18	−76.77 (13)
C2—C1—C5—C4	−0.4 (2)	C19—C20—Zr2—C18	36.88 (12)
Zr1—C1—C5—C4	66.00 (14)	C20—C16—Zr2—O3	−23.57 (14)
C2—C1—C5—Zr1	−66.39 (14)	C17—C16—Zr2—O3	−139.33 (12)
C10—C6—C7—C8	−0.8 (2)	C20—C16—Zr2—O1	−118.23 (13)
Si2—C6—C7—C8	−179.77 (15)	C17—C16—Zr2—O1	126.01 (13)
Zr1—C6—C7—C8	65.77 (15)	C17—C16—Zr2—C20	−115.76 (18)
C10—C6—C7—Zr1	−66.52 (13)	C20—C16—Zr2—C19	37.57 (12)
Si2—C6—C7—Zr1	114.46 (16)	C17—C16—Zr2—C19	−78.19 (13)
C6—C7—C8—C9	0.2 (2)	C20—C16—Zr2—C15	109.32 (13)
Zr1—C7—C8—C9	66.64 (15)	C17—C16—Zr2—C15	−6.44 (15)
C6—C7—C8—Zr1	−66.46 (15)	C20—C16—Zr2—C12	170.07 (18)
C7—C8—C9—C10	0.5 (2)	C17—C16—Zr2—C12	54.3 (3)
Zr1—C8—C9—C10	68.30 (14)	C20—C16—Zr2—C13	172.71 (12)
C7—C8—C9—Zr1	−67.82 (15)	C17—C16—Zr2—C13	56.95 (15)
C8—C9—C10—C6	−1.0 (2)	C20—C16—Zr2—C14	142.96 (12)
Zr1—C9—C10—C6	67.16 (14)	C17—C16—Zr2—C14	27.20 (14)
C8—C9—C10—Zr1	−68.12 (15)	C20—C16—Zr2—C17	115.76 (18)
C7—C6—C10—C9	1.0 (2)	C20—C16—Zr2—C11	94.31 (17)
Si2—C6—C10—C9	−179.95 (15)	C17—C16—Zr2—C11	−21.4 (2)
Zr1—C6—C10—C9	−65.91 (14)	C20—C16—Zr2—C18	77.55 (14)
C7—C6—C10—Zr1	66.96 (14)	C17—C16—Zr2—C18	−38.21 (12)
Si2—C6—C10—Zr1	−114.04 (16)	C18—C19—Zr2—O3	−158.07 (14)
C15—C11—C12—C13	−0.9 (2)	C20—C19—Zr2—O3	87.67 (13)
Zr2—C11—C12—C13	−67.38 (14)	C18—C19—Zr2—O1	107.55 (13)
C15—C11—C12—Zr2	66.44 (14)	C20—C19—Zr2—O1	−6.71 (16)
C11—C12—C13—C14	0.9 (2)	C18—C19—Zr2—C20	114.26 (19)
Zr2—C12—C13—C14	−66.59 (14)	C18—C19—Zr2—C16	76.65 (14)
C11—C12—C13—Zr2	67.51 (14)	C20—C19—Zr2—C16	−37.61 (12)
C12—C13—C14—C15	−0.5 (2)	C18—C19—Zr2—C15	−47.63 (14)
Zr2—C13—C14—C15	−67.11 (13)	C20—C19—Zr2—C15	−161.89 (13)
C12—C13—C14—Si3	−178.46 (15)	C18—C19—Zr2—C12	−80.37 (16)
Zr2—C13—C14—Si3	114.96 (16)	C20—C19—Zr2—C12	165.37 (12)
C12—C13—C14—Zr2	66.58 (14)	C18—C19—Zr2—C13	−28.9 (2)
C12—C11—C15—C14	0.6 (2)	C20—C19—Zr2—C13	−143.12 (14)
Zr2—C11—C15—C14	67.25 (14)	C18—C19—Zr2—C14	−20.57 (15)
C12—C11—C15—Zr2	−66.64 (14)	C20—C19—Zr2—C14	−134.84 (13)
C13—C14—C15—C11	−0.1 (2)	C18—C19—Zr2—C17	36.13 (12)
Si3—C14—C15—C11	177.85 (15)	C20—C19—Zr2—C17	−78.13 (14)
Zr2—C14—C15—C11	−67.55 (14)	C18—C19—Zr2—C11	−77.00 (14)
C13—C14—C15—Zr2	67.49 (13)	C20—C19—Zr2—C11	168.73 (12)
Si3—C14—C15—Zr2	−114.60 (16)	C20—C19—Zr2—C18	−114.26 (19)
C20—C16—C17—C18	1.2 (2)	C11—C15—Zr2—O3	−31.95 (15)
Zr2—C16—C17—C18	67.65 (14)	C14—C15—Zr2—O3	−147.22 (12)
C20—C16—C17—Si4	−179.68 (15)	C11—C15—Zr2—O1	89.67 (14)
Zr2—C16—C17—Si4	−113.21 (16)	C14—C15—Zr2—O1	−25.60 (16)
C20—C16—C17—Zr2	−66.46 (14)	C11—C15—Zr2—C20	−127.92 (13)
C16—C17—C18—C19	−1.0 (2)	C14—C15—Zr2—C20	116.81 (13)
Si4—C17—C18—C19	179.85 (15)	C11—C15—Zr2—C16	−165.46 (13)
Zr2—C17—C18—C19	65.33 (15)	C14—C15—Zr2—C16	79.27 (14)
C16—C17—C18—Zr2	−66.35 (13)	C11—C15—Zr2—C19	−116.35 (14)
Si4—C17—C18—Zr2	114.52 (16)	C14—C15—Zr2—C19	128.38 (13)
C17—C18—C19—C20	0.5 (2)	C11—C15—Zr2—C12	37.03 (13)
Zr2—C18—C19—C20	66.08 (14)	C14—C15—Zr2—C12	−78.24 (14)
C17—C18—C19—Zr2	−65.60 (15)	C11—C15—Zr2—C13	77.18 (14)
C17—C16—C20—C19	−0.9 (2)	C14—C15—Zr2—C13	−38.09 (12)
Zr2—C16—C20—C19	−68.59 (14)	C11—C15—Zr2—C14	115.27 (19)
C17—C16—C20—Zr2	67.66 (14)	C11—C15—Zr2—C17	−168.87 (14)
C18—C19—C20—C16	0.3 (2)	C14—C15—Zr2—C17	75.86 (13)
Zr2—C19—C20—C16	67.96 (14)	C14—C15—Zr2—C11	−115.27 (19)
C18—C19—C20—Zr2	−67.70 (15)	C11—C15—Zr2—C18	−139.91 (15)
C25—C21—C22—C23	−1.0 (2)	C14—C15—Zr2—C18	104.82 (13)
Zr3—C21—C22—C23	−67.46 (14)	C13—C12—Zr2—O3	−163.41 (13)
C25—C21—C22—Si5	179.29 (14)	C11—C12—Zr2—O3	82.24 (13)
Zr3—C21—C22—Si5	112.86 (15)	C13—C12—Zr2—O1	−65.75 (13)
C25—C21—C22—Zr3	66.43 (14)	C11—C12—Zr2—O1	179.90 (13)
C21—C22—C23—C24	0.7 (2)	C13—C12—Zr2—C20	158.9 (3)
Si5—C22—C23—C24	−179.63 (15)	C11—C12—Zr2—C20	44.5 (4)
Zr3—C22—C23—C24	−65.69 (15)	C13—C12—Zr2—C16	4.0 (3)
C21—C22—C23—Zr3	66.41 (13)	C11—C12—Zr2—C16	−110.4 (2)
Si5—C22—C23—Zr3	−113.93 (16)	C13—C12—Zr2—C19	120.49 (14)
C22—C23—C24—C25	−0.1 (2)	C11—C12—Zr2—C19	6.15 (18)
Zr3—C23—C24—C25	−65.90 (14)	C13—C12—Zr2—C15	77.42 (13)
C22—C23—C24—Zr3	65.77 (15)	C11—C12—Zr2—C15	−36.93 (12)
C22—C21—C25—C24	1.0 (2)	C11—C12—Zr2—C13	−114.35 (19)
Zr3—C21—C25—C24	68.45 (14)	C13—C12—Zr2—C14	36.61 (12)
C22—C21—C25—Zr3	−67.48 (14)	C11—C12—Zr2—C14	−77.74 (14)
C23—C24—C25—C21	−0.5 (2)	C13—C12—Zr2—C17	40.37 (17)
Zr3—C24—C25—C21	−67.87 (14)	C11—C12—Zr2—C17	−73.97 (16)
C23—C24—C25—Zr3	67.35 (14)	C13—C12—Zr2—C11	114.35 (19)
C30—C26—C27—C28	−0.5 (2)	C13—C12—Zr2—C18	81.06 (15)
Zr3—C26—C27—C28	66.23 (14)	C11—C12—Zr2—C18	−33.29 (16)
C30—C26—C27—Zr3	−66.69 (14)	C12—C13—Zr2—O3	18.74 (15)
C26—C27—C28—C29	−0.1 (2)	C14—C13—Zr2—O3	134.48 (12)
Zr3—C27—C28—C29	66.20 (14)	C12—C13—Zr2—O1	112.26 (13)
C26—C27—C28—Zr3	−66.32 (14)	C14—C13—Zr2—O1	−132.00 (13)
C27—C28—C29—C30	0.6 (2)	C12—C13—Zr2—C20	−167.11 (18)
Zr3—C28—C29—C30	67.97 (13)	C14—C13—Zr2—C20	−51.4 (3)
C27—C28—C29—Si6	−179.77 (15)	C12—C13—Zr2—C16	−178.25 (12)
Zr3—C28—C29—Si6	−112.45 (15)	C14—C13—Zr2—C16	−62.51 (15)
C27—C28—C29—Zr3	−67.33 (14)	C12—C13—Zr2—C19	−101.23 (16)
C27—C26—C30—C29	0.9 (2)	C14—C13—Zr2—C19	14.5 (2)
Zr3—C26—C30—C29	−66.72 (14)	C12—C13—Zr2—C15	−77.28 (14)
C27—C26—C30—Zr3	67.59 (14)	C14—C13—Zr2—C15	38.46 (12)
C28—C29—C30—C26	−0.9 (2)	C14—C13—Zr2—C12	115.74 (19)
Si6—C29—C30—C26	179.49 (15)	C12—C13—Zr2—C14	−115.74 (19)
Zr3—C29—C30—C26	67.46 (15)	C12—C13—Zr2—C17	−150.52 (12)
C28—C29—C30—Zr3	−68.39 (13)	C14—C13—Zr2—C17	−34.78 (14)
Si6—C29—C30—Zr3	112.04 (16)	C12—C13—Zr2—C11	−37.14 (13)
C2—C3—Si1—C40	106.3 (2)	C14—C13—Zr2—C11	78.60 (14)
C4—C3—Si1—C40	−74.2 (2)	C12—C13—Zr2—C18	−117.46 (13)
Zr1—C3—Si1—C40	−163.18 (12)	C14—C13—Zr2—C18	−1.71 (15)
C2—C3—Si1—C41	−13.1 (2)	C13—C14—Zr2—O3	−65.67 (15)
C4—C3—Si1—C41	166.43 (19)	C15—C14—Zr2—O3	46.70 (16)
Zr1—C3—Si1—C41	77.42 (14)	Si3—C14—Zr2—O3	170.81 (8)
C2—C3—Si1—Si2	−131.27 (17)	C13—C14—Zr2—O1	48.24 (13)
C4—C3—Si1—Si2	48.27 (18)	C15—C14—Zr2—O1	160.61 (12)
Zr1—C3—Si1—Si2	−40.73 (10)	Si3—C14—Zr2—O1	−75.28 (11)
C10—C6—Si2—C42	−28.2 (2)	C13—C14—Zr2—C20	158.21 (12)
C7—C6—Si2—C42	150.58 (19)	C15—C14—Zr2—C20	−89.42 (15)
Zr1—C6—Si2—C42	−118.68 (13)	Si3—C14—Zr2—C20	34.69 (16)
C10—C6—Si2—C43	−148.61 (19)	C13—C14—Zr2—C16	132.38 (13)
C7—C6—Si2—C43	30.2 (2)	C15—C14—Zr2—C16	−115.25 (13)
Zr1—C6—Si2—C43	120.92 (12)	Si3—C14—Zr2—C16	8.86 (13)
C10—C6—Si2—Si1	91.96 (18)	C13—C14—Zr2—C19	−170.96 (12)
C7—C6—Si2—Si1	−89.24 (18)	C15—C14—Zr2—C19	−58.59 (14)
Zr1—C6—Si2—Si1	1.49 (11)	Si3—C14—Zr2—C19	65.53 (13)
C3—Si1—Si2—C42	141.35 (11)	C13—C14—Zr2—C15	−112.37 (18)
C40—Si1—Si2—C42	−98.81 (13)	Si3—C14—Zr2—C15	124.12 (18)
C41—Si1—Si2—C42	25.47 (14)	C13—C14—Zr2—C12	−36.19 (12)
C3—Si1—Si2—C6	23.54 (10)	C15—C14—Zr2—C12	76.18 (14)
C40—Si1—Si2—C6	143.37 (12)	Si3—C14—Zr2—C12	−159.71 (15)
C41—Si1—Si2—C6	−92.34 (12)	C15—C14—Zr2—C13	112.37 (18)
C3—Si1—Si2—C43	−92.92 (12)	Si3—C14—Zr2—C13	−123.51 (18)
C40—Si1—Si2—C43	26.91 (14)	C13—C14—Zr2—C17	146.57 (13)
C41—Si1—Si2—C43	151.20 (14)	C15—C14—Zr2—C17	−101.06 (13)
C13—C14—Si3—C45	15.5 (2)	Si3—C14—Zr2—C17	23.05 (11)
C15—C14—Si3—C45	−162.00 (19)	C13—C14—Zr2—C11	−76.07 (14)
Zr2—C14—Si3—C45	106.91 (13)	C15—C14—Zr2—C11	36.30 (12)
C13—C14—Si3—C44	134.99 (19)	Si3—C14—Zr2—C11	160.41 (15)
C15—C14—Si3—C44	−42.5 (2)	C13—C14—Zr2—C18	178.45 (13)
Zr2—C14—Si3—C44	−133.60 (12)	C15—C14—Zr2—C18	−69.18 (13)
C13—C14—Si3—Si4	−104.20 (17)	Si3—C14—Zr2—C18	54.94 (11)
C15—C14—Si3—Si4	78.31 (18)	C16—C17—Zr2—O3	56.83 (15)
Zr2—C14—Si3—Si4	−12.79 (11)	C18—C17—Zr2—O3	−55.41 (15)
C16—C17—Si4—C46	−118.7 (2)	Si4—C17—Zr2—O3	−179.71 (8)
C18—C17—Si4—C46	60.3 (2)	C16—C17—Zr2—O1	−54.13 (13)
Zr2—C17—Si4—C46	152.05 (14)	C18—C17—Zr2—O1	−166.37 (12)
C16—C17—Si4—C47	1.7 (2)	Si4—C17—Zr2—O1	69.33 (11)
C18—C17—Si4—C47	−179.4 (2)	C16—C17—Zr2—C20	36.44 (12)
Zr2—C17—Si4—C47	−87.60 (15)	C18—C17—Zr2—C20	−75.80 (13)
C16—C17—Si4—Si3	118.24 (17)	Si4—C17—Zr2—C20	159.90 (15)
C18—C17—Si4—Si3	−62.80 (19)	C18—C17—Zr2—C16	−112.23 (18)
Zr2—C17—Si4—Si3	28.99 (11)	Si4—C17—Zr2—C16	123.46 (18)
C45—Si3—Si4—C17	−126.52 (12)	C16—C17—Zr2—C19	76.59 (14)
C14—Si3—Si4—C17	−9.62 (10)	C18—C17—Zr2—C19	−35.65 (12)
C44—Si3—Si4—C17	108.81 (11)	Si4—C17—Zr2—C19	−159.95 (15)
C45—Si3—Si4—C46	113.29 (16)	C16—C17—Zr2—C15	174.42 (13)
C14—Si3—Si4—C46	−129.81 (15)	C18—C17—Zr2—C15	62.19 (13)
C44—Si3—Si4—C46	−11.38 (16)	Si4—C17—Zr2—C15	−62.12 (11)
C45—Si3—Si4—C47	−11.32 (15)	C16—C17—Zr2—C12	−157.19 (12)
C14—Si3—Si4—C47	105.58 (14)	C18—C17—Zr2—C12	90.57 (15)
C44—Si3—Si4—C47	−135.99 (15)	Si4—C17—Zr2—C12	−33.73 (16)
C21—C22—Si5—C49	131.19 (19)	C16—C17—Zr2—C13	−136.46 (13)
C23—C22—Si5—C49	−48.4 (2)	C18—C17—Zr2—C13	111.31 (13)
Zr3—C22—Si5—C49	−140.63 (13)	Si4—C17—Zr2—C13	−12.99 (13)
C21—C22—Si5—C48	11.7 (2)	C16—C17—Zr2—C14	−154.16 (13)
C23—C22—Si5—C48	−167.9 (2)	C18—C17—Zr2—C14	93.60 (13)
Zr3—C22—Si5—C48	99.87 (14)	Si4—C17—Zr2—C14	−30.70 (11)
C21—C22—Si5—Si6	−107.50 (16)	C16—C17—Zr2—C11	167.82 (12)
C23—C22—Si5—Si6	72.90 (19)	C18—C17—Zr2—C11	55.59 (14)
Zr3—C22—Si5—Si6	−19.31 (11)	Si4—C17—Zr2—C11	−68.71 (13)
C28—C29—Si6—C50	−121.20 (19)	C16—C17—Zr2—C18	112.23 (18)
C30—C29—Si6—C50	58.3 (2)	Si4—C17—Zr2—C18	−124.30 (18)
Zr3—C29—Si6—C50	148.57 (13)	C15—C11—Zr2—O3	150.17 (14)
C28—C29—Si6—C51	−0.7 (2)	C12—C11—Zr2—O3	−95.27 (13)
C30—C29—Si6—C51	178.74 (19)	C15—C11—Zr2—O1	−114.68 (13)
Zr3—C29—Si6—C51	−90.98 (14)	C12—C11—Zr2—O1	−0.12 (15)
C28—C29—Si6—Si5	116.17 (17)	C15—C11—Zr2—C20	78.66 (16)
C30—C29—Si6—Si5	−64.34 (19)	C12—C11—Zr2—C20	−166.77 (12)
Zr3—C29—Si6—Si5	25.94 (11)	C15—C11—Zr2—C16	25.5 (2)
C22—Si5—Si6—C50	−124.48 (12)	C12—C11—Zr2—C16	140.07 (15)
C49—Si5—Si6—C50	−6.38 (16)	C15—C11—Zr2—C19	69.65 (14)
C48—Si5—Si6—C50	119.21 (14)	C12—C11—Zr2—C19	−175.79 (13)
C22—Si5—Si6—C51	111.07 (12)	C12—C11—Zr2—C15	114.56 (19)
C49—Si5—Si6—C51	−130.83 (15)	C15—C11—Zr2—C12	−114.56 (19)
C48—Si5—Si6—C51	−5.24 (14)	C15—C11—Zr2—C13	−77.51 (14)
C22—Si5—Si6—C29	−4.09 (10)	C12—C11—Zr2—C13	37.06 (12)
C49—Si5—Si6—C29	114.02 (13)	C15—C11—Zr2—C14	−37.09 (12)
C48—Si5—Si6—C29	−120.39 (12)	C12—C11—Zr2—C14	77.48 (14)
Zr2—O1—Zr1—O2	2.06 (13)	C15—C11—Zr2—C17	12.73 (16)
Zr2—O1—Zr1—C9	−88.88 (13)	C12—C11—Zr2—C17	127.30 (13)
Zr2—O1—Zr1—C4	142.66 (12)	C15—C11—Zr2—C18	38.80 (14)
Zr2—O1—Zr1—C3	110.84 (13)	C12—C11—Zr2—C18	153.36 (13)
Zr2—O1—Zr1—C8	−120.71 (13)	C19—C18—Zr2—O3	23.56 (14)
Zr2—O1—Zr1—C10	−79.64 (13)	C17—C18—Zr2—O3	140.25 (12)
Zr2—O1—Zr1—C7	−136.47 (12)	C19—C18—Zr2—O1	−98.90 (14)
Zr2—O1—Zr1—C2	85.63 (13)	C17—C18—Zr2—O1	17.78 (16)
Zr2—O1—Zr1—C5	131.99 (13)	C19—C18—Zr2—C20	−37.49 (13)
Zr2—O1—Zr1—C6	−112.55 (13)	C17—C18—Zr2—C20	79.19 (13)
Zr2—O1—Zr1—C1	100.64 (13)	C19—C18—Zr2—C16	−78.22 (14)
Zr3—O2—Zr1—O1	1.20 (12)	C17—C18—Zr2—C16	38.46 (12)
Zr3—O2—Zr1—C9	88.27 (12)	C17—C18—Zr2—C19	116.69 (19)
Zr3—O2—Zr1—C4	−131.62 (11)	C19—C18—Zr2—C15	130.09 (14)
Zr3—O2—Zr1—C3	−136.99 (12)	C17—C18—Zr2—C15	−113.22 (13)
Zr3—O2—Zr1—C8	88.21 (13)	C19—C18—Zr2—C12	127.11 (13)
Zr3—O2—Zr1—C10	117.62 (12)	C17—C18—Zr2—C12	−116.21 (13)
Zr3—O2—Zr1—C7	126.79 (12)	C19—C18—Zr2—C13	162.05 (13)
Zr3—O2—Zr1—C2	−109.38 (12)	C17—C18—Zr2—C13	−81.26 (13)
Zr3—O2—Zr1—C5	−90.61 (13)	C19—C18—Zr2—C14	161.15 (14)
Zr3—O2—Zr1—C6	140.80 (12)	C17—C18—Zr2—C14	−82.17 (13)
Zr3—O2—Zr1—C1	−83.82 (12)	C19—C18—Zr2—C17	−116.69 (19)
C10—C9—Zr1—O1	164.25 (13)	C19—C18—Zr2—C11	110.25 (13)
C8—C9—Zr1—O1	−82.00 (13)	C17—C18—Zr2—C11	−133.06 (13)
C10—C9—Zr1—O2	66.34 (13)	Zr1—O2—Zr3—O3	−2.45 (12)
C8—C9—Zr1—O2	−179.91 (13)	Zr1—O2—Zr3—C25	90.30 (12)
C10—C9—Zr1—C4	−75.78 (15)	Zr1—O2—Zr3—C21	78.38 (13)
C8—C9—Zr1—C4	37.97 (16)	Zr1—O2—Zr3—C24	122.47 (12)
C10—C9—Zr1—C3	−34.90 (17)	Zr1—O2—Zr3—C30	−143.35 (11)
C8—C9—Zr1—C3	78.85 (15)	Zr1—O2—Zr3—C29	−114.18 (12)
C10—C9—Zr1—C8	−113.75 (19)	Zr1—O2—Zr3—C22	107.89 (12)
C8—C9—Zr1—C10	113.75 (19)	Zr1—O2—Zr3—C28	−85.90 (13)
C10—C9—Zr1—C7	−76.73 (13)	Zr1—O2—Zr3—C23	135.36 (11)
C8—C9—Zr1—C7	37.01 (13)	Zr1—O2—Zr3—C26	−130.59 (12)
C10—C9—Zr1—C2	0.0 (3)	Zr1—O2—Zr3—C27	−99.04 (12)
C8—C9—Zr1—C2	113.8 (2)	Zr2—O3—Zr3—O2	1.73 (13)
C10—C9—Zr1—C5	−114.94 (14)	Zr2—O3—Zr3—C25	−82.34 (12)
C8—C9—Zr1—C5	−1.19 (18)	Zr2—O3—Zr3—C21	−110.52 (12)
C10—C9—Zr1—C6	−36.36 (12)	Zr2—O3—Zr3—C24	−85.61 (13)
C8—C9—Zr1—C6	77.39 (14)	Zr2—O3—Zr3—C30	133.44 (11)
C10—C9—Zr1—C1	−150.0 (3)	Zr2—O3—Zr3—C29	141.39 (12)
C8—C9—Zr1—C1	−36.2 (4)	Zr2—O3—Zr3—C22	−135.65 (12)
C5—C4—Zr1—O1	−20.79 (15)	Zr2—O3—Zr3—C28	114.84 (12)
C3—C4—Zr1—O1	−136.18 (12)	Zr2—O3—Zr3—C23	−125.11 (12)
C5—C4—Zr1—O2	105.35 (14)	Zr2—O3—Zr3—C26	92.76 (13)
C3—C4—Zr1—O2	−10.04 (16)	Zr2—O3—Zr3—C27	88.26 (12)
C5—C4—Zr1—C9	−126.71 (14)	C21—C25—Zr3—O2	−158.97 (13)
C3—C4—Zr1—C9	117.90 (13)	C24—C25—Zr3—O2	87.11 (12)
C5—C4—Zr1—C3	115.40 (19)	C21—C25—Zr3—O3	−61.05 (13)
C5—C4—Zr1—C8	−107.48 (14)	C24—C25—Zr3—O3	−174.97 (12)
C3—C4—Zr1—C8	137.13 (13)	C24—C25—Zr3—C21	−113.92 (18)
C5—C4—Zr1—C10	−160.28 (13)	C21—C25—Zr3—C24	113.92 (18)
C3—C4—Zr1—C10	84.33 (13)	C21—C25—Zr3—C30	85.39 (14)
C5—C4—Zr1—C7	−125.91 (14)	C24—C25—Zr3—C30	−28.53 (15)
C3—C4—Zr1—C7	118.69 (13)	C21—C25—Zr3—C29	44.73 (16)
C5—C4—Zr1—C2	77.03 (14)	C24—C25—Zr3—C29	−69.19 (15)
C3—C4—Zr1—C2	−38.36 (12)	C21—C25—Zr3—C22	36.68 (12)
C3—C4—Zr1—C5	−115.40 (19)	C24—C25—Zr3—C22	−77.24 (13)
C5—C4—Zr1—C6	−157.58 (14)	C21—C25—Zr3—C28	9.7 (3)
C3—C4—Zr1—C6	87.03 (13)	C24—C25—Zr3—C28	−104.2 (2)
C5—C4—Zr1—C1	37.00 (13)	C21—C25—Zr3—C23	77.06 (13)
C3—C4—Zr1—C1	−78.39 (13)	C24—C25—Zr3—C23	−36.86 (12)
C2—C3—Zr1—O1	−48.80 (15)	C21—C25—Zr3—C26	124.67 (13)
C4—C3—Zr1—O1	63.02 (15)	C24—C25—Zr3—C26	10.75 (17)
Si1—C3—Zr1—O1	−173.05 (8)	C21—C25—Zr3—C27	160.0 (2)
C2—C3—Zr1—O2	60.32 (12)	C24—C25—Zr3—C27	46.1 (3)
C4—C3—Zr1—O2	172.14 (12)	C25—C21—Zr3—O2	22.85 (14)
Si1—C3—Zr1—O2	−63.94 (10)	C22—C21—Zr3—O2	138.38 (11)
C2—C3—Zr1—C9	159.16 (12)	C25—C21—Zr3—O3	116.92 (13)
C4—C3—Zr1—C9	−89.02 (15)	C22—C21—Zr3—O3	−127.55 (12)
Si1—C3—Zr1—C9	34.90 (15)	C22—C21—Zr3—C25	115.52 (18)
C2—C3—Zr1—C4	−111.82 (18)	C25—C21—Zr3—C24	−37.49 (12)
Si1—C3—Zr1—C4	123.92 (17)	C22—C21—Zr3—C24	78.03 (13)
C2—C3—Zr1—C8	−163.54 (12)	C25—C21—Zr3—C30	−114.14 (13)
C4—C3—Zr1—C8	−51.72 (15)	C22—C21—Zr3—C30	1.38 (14)
Si1—C3—Zr1—C8	72.20 (12)	C25—C21—Zr3—C29	−148.01 (12)
C2—C3—Zr1—C10	140.91 (12)	C22—C21—Zr3—C29	−32.48 (13)
C4—C3—Zr1—C10	−107.27 (13)	C25—C21—Zr3—C22	−115.52 (18)
Si1—C3—Zr1—C10	16.65 (12)	C25—C21—Zr3—C28	−176.08 (12)
C2—C3—Zr1—C7	−169.01 (13)	C22—C21—Zr3—C28	−60.56 (15)
C4—C3—Zr1—C7	−57.18 (13)	C25—C21—Zr3—C23	−77.43 (14)
Si1—C3—Zr1—C7	66.74 (11)	C22—C21—Zr3—C23	38.09 (12)
C4—C3—Zr1—C2	111.82 (18)	C25—C21—Zr3—C26	−96.94 (16)
Si1—C3—Zr1—C2	−124.26 (17)	C22—C21—Zr3—C26	18.58 (19)
C2—C3—Zr1—C5	−75.64 (13)	C25—C21—Zr3—C27	−165.1 (2)
C4—C3—Zr1—C5	36.19 (12)	C22—C21—Zr3—C27	−49.5 (3)
Si1—C3—Zr1—C5	160.11 (14)	C23—C24—Zr3—O2	156.61 (13)
C2—C3—Zr1—C6	160.33 (13)	C25—C24—Zr3—O2	−89.06 (12)
C4—C3—Zr1—C6	−87.84 (13)	C23—C24—Zr3—O3	−108.16 (13)
Si1—C3—Zr1—C6	36.08 (10)	C25—C24—Zr3—O3	6.17 (15)
C2—C3—Zr1—C1	−35.99 (12)	C23—C24—Zr3—C25	−114.33 (18)
C4—C3—Zr1—C1	75.83 (13)	C23—C24—Zr3—C21	−76.89 (14)
Si1—C3—Zr1—C1	−160.25 (14)	C25—C24—Zr3—C21	37.44 (12)
C7—C8—Zr1—O1	−150.79 (14)	C23—C24—Zr3—C30	42.65 (14)
C9—C8—Zr1—O1	95.17 (13)	C25—C24—Zr3—C30	156.98 (12)
C7—C8—Zr1—O2	114.15 (13)	C23—C24—Zr3—C29	17.13 (15)
C9—C8—Zr1—O2	0.11 (16)	C25—C24—Zr3—C29	131.45 (12)
C7—C8—Zr1—C9	114.0 (2)	C23—C24—Zr3—C22	−36.41 (12)
C7—C8—Zr1—C4	−35.53 (14)	C25—C24—Zr3—C22	77.92 (13)
C9—C8—Zr1—C4	−149.57 (13)	C23—C24—Zr3—C28	31.4 (2)
C7—C8—Zr1—C3	−10.52 (16)	C25—C24—Zr3—C28	145.73 (13)
C9—C8—Zr1—C3	−124.56 (13)	C25—C24—Zr3—C23	114.33 (18)
C7—C8—Zr1—C10	76.68 (14)	C23—C24—Zr3—C26	73.17 (14)
C9—C8—Zr1—C10	−37.36 (12)	C25—C24—Zr3—C26	−172.50 (12)
C9—C8—Zr1—C7	−114.0 (2)	C23—C24—Zr3—C27	80.79 (16)
C7—C8—Zr1—C2	−28.3 (2)	C25—C24—Zr3—C27	−164.88 (12)
C9—C8—Zr1—C2	−142.30 (15)	C26—C30—Zr3—O2	25.05 (15)
C7—C8—Zr1—C5	−66.79 (15)	C29—C30—Zr3—O2	140.69 (11)
C9—C8—Zr1—C5	179.18 (13)	C26—C30—Zr3—O3	−100.68 (14)
C7—C8—Zr1—C6	36.36 (13)	C29—C30—Zr3—O3	14.97 (15)
C9—C8—Zr1—C6	−77.68 (14)	C26—C30—Zr3—C25	125.17 (13)
C7—C8—Zr1—C1	−77.36 (16)	C29—C30—Zr3—C25	−119.18 (12)
C9—C8—Zr1—C1	168.60 (12)	C26—C30—Zr3—C21	161.56 (12)
C9—C10—Zr1—O1	−17.76 (15)	C29—C30—Zr3—C21	−82.79 (13)
C6—C10—Zr1—O1	−133.81 (12)	C26—C30—Zr3—C24	110.43 (13)
C9—C10—Zr1—O2	−111.12 (13)	C29—C30—Zr3—C24	−133.92 (12)
C6—C10—Zr1—O2	132.83 (13)	C26—C30—Zr3—C29	−115.65 (19)
C6—C10—Zr1—C9	−116.05 (19)	C26—C30—Zr3—C22	162.30 (14)
C9—C10—Zr1—C4	121.12 (13)	C29—C30—Zr3—C22	−82.06 (12)
C6—C10—Zr1—C4	5.07 (15)	C26—C30—Zr3—C28	−77.13 (14)
C9—C10—Zr1—C3	154.62 (12)	C29—C30—Zr3—C28	38.52 (12)
C6—C10—Zr1—C3	38.57 (14)	C26—C30—Zr3—C23	132.04 (14)
C9—C10—Zr1—C8	37.67 (13)	C29—C30—Zr3—C23	−112.31 (13)
C6—C10—Zr1—C8	−78.38 (14)	C29—C30—Zr3—C26	115.65 (19)
C9—C10—Zr1—C7	77.76 (14)	C26—C30—Zr3—C27	−37.23 (13)
C6—C10—Zr1—C7	−38.28 (12)	C29—C30—Zr3—C27	78.42 (13)
C9—C10—Zr1—C2	−179.98 (12)	C28—C29—Zr3—O2	55.01 (15)
C6—C10—Zr1—C2	63.97 (15)	C30—C29—Zr3—O2	−56.79 (15)
C9—C10—Zr1—C5	104.63 (15)	Si6—C29—Zr3—O2	178.31 (8)
C6—C10—Zr1—C5	−11.41 (19)	C28—C29—Zr3—O3	−56.62 (13)
C9—C10—Zr1—C6	116.05 (19)	C30—C29—Zr3—O3	−168.42 (12)
C9—C10—Zr1—C1	161.46 (18)	Si6—C29—Zr3—O3	66.68 (11)
C6—C10—Zr1—C1	45.4 (3)	C28—C29—Zr3—C25	−160.42 (12)
C8—C7—Zr1—O1	31.15 (15)	C30—C29—Zr3—C25	87.78 (14)
C6—C7—Zr1—O1	147.47 (12)	Si6—C29—Zr3—C25	−37.12 (15)
C8—C7—Zr1—O2	−89.56 (15)	C28—C29—Zr3—C21	−137.21 (12)
C6—C7—Zr1—O2	26.76 (15)	C30—C29—Zr3—C21	111.00 (13)
C8—C7—Zr1—C9	−37.64 (13)	Si6—C29—Zr3—C21	−13.91 (12)
C6—C7—Zr1—C9	78.68 (13)	C28—C29—Zr3—C24	165.64 (12)
C8—C7—Zr1—C4	143.18 (15)	C30—C29—Zr3—C24	53.85 (14)
C6—C7—Zr1—C4	−100.50 (13)	Si6—C29—Zr3—C24	−71.06 (12)
C8—C7—Zr1—C3	171.01 (14)	C28—C29—Zr3—C30	111.80 (18)
C6—C7—Zr1—C3	−72.67 (13)	Si6—C29—Zr3—C30	−124.90 (18)
C6—C7—Zr1—C8	116.32 (19)	C28—C29—Zr3—C22	−153.92 (13)
C8—C7—Zr1—C10	−78.10 (15)	C30—C29—Zr3—C22	94.29 (13)
C6—C7—Zr1—C10	38.22 (12)	Si6—C29—Zr3—C22	−30.62 (10)
C8—C7—Zr1—C2	164.25 (13)	C30—C29—Zr3—C28	−111.80 (18)
C6—C7—Zr1—C2	−79.43 (14)	Si6—C29—Zr3—C28	123.30 (18)
C8—C7—Zr1—C5	118.19 (14)	C28—C29—Zr3—C23	174.48 (13)
C6—C7—Zr1—C5	−125.49 (13)	C30—C29—Zr3—C23	62.69 (13)
C8—C7—Zr1—C6	−116.32 (19)	Si6—C29—Zr3—C23	−62.22 (11)
C8—C7—Zr1—C1	127.20 (14)	C28—C29—Zr3—C26	75.72 (13)
C6—C7—Zr1—C1	−116.48 (13)	C30—C29—Zr3—C26	−36.08 (12)
C1—C2—Zr1—O1	29.74 (14)	Si6—C29—Zr3—C26	−160.98 (14)
C3—C2—Zr1—O1	145.61 (11)	C28—C29—Zr3—C27	35.87 (12)
C1—C2—Zr1—O2	124.87 (13)	C30—C29—Zr3—C27	−75.92 (13)
C3—C2—Zr1—O2	−119.25 (12)	Si6—C29—Zr3—C27	159.17 (14)
C1—C2—Zr1—C9	−167.24 (19)	C21—C22—Zr3—O2	−58.61 (15)
C3—C2—Zr1—C9	−51.4 (3)	C23—C22—Zr3—O2	54.05 (15)
C1—C2—Zr1—C4	−77.07 (14)	Si5—C22—Zr3—O2	−179.88 (8)
C3—C2—Zr1—C4	38.81 (12)	C21—C22—Zr3—O3	51.70 (12)
C1—C2—Zr1—C3	−115.88 (19)	C23—C22—Zr3—O3	164.36 (12)
C1—C2—Zr1—C8	−86.19 (19)	Si5—C22—Zr3—O3	−69.57 (11)
C3—C2—Zr1—C8	29.7 (2)	C21—C22—Zr3—C25	−36.57 (12)
C1—C2—Zr1—C10	−167.21 (12)	C23—C22—Zr3—C25	76.09 (13)
C3—C2—Zr1—C10	−51.34 (15)	Si5—C22—Zr3—C25	−157.85 (14)
C1—C2—Zr1—C7	−102.98 (14)	C23—C22—Zr3—C21	112.66 (18)
C3—C2—Zr1—C7	12.89 (15)	Si5—C22—Zr3—C21	−121.27 (17)
C1—C2—Zr1—C5	−36.88 (13)	C21—C22—Zr3—C24	−76.84 (13)
C3—C2—Zr1—C5	79.00 (13)	C23—C22—Zr3—C24	35.82 (12)
C1—C2—Zr1—C6	−136.73 (13)	Si5—C22—Zr3—C24	161.89 (14)
C3—C2—Zr1—C6	−20.86 (14)	C21—C22—Zr3—C30	−178.77 (12)
C3—C2—Zr1—C1	115.88 (19)	C23—C22—Zr3—C30	−66.11 (13)
C4—C5—Zr1—O1	161.34 (13)	Si5—C22—Zr3—C30	59.96 (11)
C1—C5—Zr1—O1	−84.28 (13)	C21—C22—Zr3—C29	149.20 (13)
C4—C5—Zr1—O2	−101.39 (14)	C23—C22—Zr3—C29	−98.14 (13)
C1—C5—Zr1—O2	12.98 (16)	Si5—C22—Zr3—C29	27.93 (10)
C4—C5—Zr1—C9	80.29 (16)	C21—C22—Zr3—C28	134.84 (12)
C1—C5—Zr1—C9	−165.33 (12)	C23—C22—Zr3—C28	−112.50 (13)
C1—C5—Zr1—C4	114.37 (19)	Si5—C22—Zr3—C28	13.57 (12)
C4—C5—Zr1—C3	−37.09 (12)	C21—C22—Zr3—C23	−112.66 (18)
C1—C5—Zr1—C3	77.29 (14)	Si5—C22—Zr3—C23	126.07 (18)
C4—C5—Zr1—C8	79.63 (14)	C21—C22—Zr3—C26	−168.70 (11)
C1—C5—Zr1—C8	−165.99 (13)	C23—C22—Zr3—C26	−56.04 (14)
C4—C5—Zr1—C10	30.09 (19)	Si5—C22—Zr3—C26	70.02 (12)
C1—C5—Zr1—C10	144.46 (13)	C21—C22—Zr3—C27	159.95 (12)
C4—C5—Zr1—C7	50.97 (14)	C23—C22—Zr3—C27	−87.39 (15)
C1—C5—Zr1—C7	165.34 (13)	Si5—C22—Zr3—C27	38.67 (15)
C4—C5—Zr1—C2	−77.55 (14)	C27—C28—Zr3—O2	−25.96 (14)
C1—C5—Zr1—C2	36.82 (12)	C29—C28—Zr3—O2	−141.84 (11)
C4—C5—Zr1—C6	23.70 (15)	C27—C28—Zr3—O3	−120.93 (13)
C1—C5—Zr1—C6	138.08 (13)	C29—C28—Zr3—O3	123.20 (13)
C4—C5—Zr1—C1	−114.37 (19)	C27—C28—Zr3—C25	166.3 (2)
C10—C6—Zr1—O1	66.34 (15)	C29—C28—Zr3—C25	50.5 (3)
C7—C6—Zr1—O1	−46.02 (15)	C27—C28—Zr3—C21	173.07 (12)
Si2—C6—Zr1—O1	−169.39 (8)	C29—C28—Zr3—C21	57.19 (15)
C10—C6—Zr1—O2	−47.16 (13)	C27—C28—Zr3—C24	91.45 (17)
C7—C6—Zr1—O2	−159.52 (12)	C29—C28—Zr3—C24	−24.4 (2)
Si2—C6—Zr1—O2	77.11 (11)	C27—C28—Zr3—C30	77.21 (14)
C10—C6—Zr1—C9	36.21 (13)	C29—C28—Zr3—C30	−38.67 (12)
C7—C6—Zr1—C9	−76.16 (14)	C27—C28—Zr3—C29	115.88 (19)
Si2—C6—Zr1—C9	160.48 (15)	C27—C28—Zr3—C22	143.56 (13)
C10—C6—Zr1—C4	−175.28 (14)	C29—C28—Zr3—C22	27.68 (14)
C7—C6—Zr1—C4	72.36 (13)	C27—C28—Zr3—C23	109.57 (13)
Si2—C6—Zr1—C4	−51.01 (11)	C29—C28—Zr3—C23	−6.31 (15)
C10—C6—Zr1—C3	−142.73 (13)	C27—C28—Zr3—C26	37.07 (13)
C7—C6—Zr1—C3	104.90 (13)	C29—C28—Zr3—C26	−78.81 (13)
Si2—C6—Zr1—C3	−18.46 (11)	C29—C28—Zr3—C27	−115.88 (19)
C10—C6—Zr1—C8	76.51 (14)	C24—C23—Zr3—O2	−25.13 (14)
C7—C6—Zr1—C8	−35.85 (12)	C22—C23—Zr3—O2	−141.45 (12)
Si2—C6—Zr1—C8	−159.22 (15)	C24—C23—Zr3—O3	96.31 (14)
C7—C6—Zr1—C10	−112.36 (18)	C22—C23—Zr3—O3	−20.02 (16)
Si2—C6—Zr1—C10	124.27 (19)	C24—C23—Zr3—C25	37.50 (12)
C10—C6—Zr1—C7	112.36 (18)	C22—C23—Zr3—C25	−78.83 (13)
Si2—C6—Zr1—C7	−123.37 (18)	C24—C23—Zr3—C21	78.14 (14)
C10—C6—Zr1—C2	−131.78 (13)	C22—C23—Zr3—C21	−38.19 (11)
C7—C6—Zr1—C2	115.85 (13)	C22—C23—Zr3—C24	−116.32 (19)
Si2—C6—Zr1—C2	−7.51 (13)	C24—C23—Zr3—C30	−135.46 (14)
C10—C6—Zr1—C5	172.49 (12)	C22—C23—Zr3—C30	108.22 (13)
C7—C6—Zr1—C5	60.12 (14)	C24—C23—Zr3—C29	−164.86 (13)
Si2—C6—Zr1—C5	−63.24 (12)	C22—C23—Zr3—C29	78.82 (13)
C10—C6—Zr1—C1	−160.02 (12)	C24—C23—Zr3—C22	116.32 (19)
C7—C6—Zr1—C1	87.62 (15)	C24—C23—Zr3—C28	−161.53 (12)
Si2—C6—Zr1—C1	−35.74 (15)	C22—C23—Zr3—C28	82.15 (14)
C2—C1—Zr1—O1	−152.33 (13)	C24—C23—Zr3—C26	−112.62 (13)
C5—C1—Zr1—O1	92.94 (13)	C22—C23—Zr3—C26	131.06 (13)
C2—C1—Zr1—O2	−55.23 (13)	C24—C23—Zr3—C27	−125.21 (13)
C5—C1—Zr1—O2	−169.96 (13)	C22—C23—Zr3—C27	118.47 (13)
C2—C1—Zr1—C9	161.6 (3)	C30—C26—Zr3—O2	−157.27 (13)
C5—C1—Zr1—C9	46.9 (4)	C27—C26—Zr3—O2	88.54 (13)
C2—C1—Zr1—C4	77.62 (14)	C30—C26—Zr3—O3	105.60 (14)
C5—C1—Zr1—C4	−37.11 (12)	C27—C26—Zr3—O3	−8.59 (16)
C2—C1—Zr1—C3	36.63 (12)	C30—C26—Zr3—C25	−81.69 (16)
C5—C1—Zr1—C3	−78.10 (14)	C27—C26—Zr3—C25	164.13 (12)
C2—C1—Zr1—C8	134.50 (13)	C30—C26—Zr3—C21	−30.32 (19)
C5—C1—Zr1—C8	19.77 (17)	C27—C26—Zr3—C21	−144.50 (13)
C2—C1—Zr1—C10	28.4 (3)	C30—C26—Zr3—C24	−75.79 (14)
C5—C1—Zr1—C10	−86.4 (2)	C27—C26—Zr3—C24	170.03 (12)
C2—C1—Zr1—C7	97.21 (14)	C27—C26—Zr3—C30	−114.19 (19)
C5—C1—Zr1—C7	−17.52 (16)	C30—C26—Zr3—C29	36.87 (12)
C5—C1—Zr1—C2	−114.73 (19)	C27—C26—Zr3—C29	−77.32 (13)
C2—C1—Zr1—C5	114.73 (19)	C30—C26—Zr3—C22	−19.56 (15)
C2—C1—Zr1—C6	58.30 (16)	C27—C26—Zr3—C22	−133.75 (12)
C5—C1—Zr1—C6	−56.43 (16)	C30—C26—Zr3—C28	77.48 (14)
Zr3—O3—Zr2—O1	−0.11 (13)	C27—C26—Zr3—C28	−36.71 (12)
Zr3—O3—Zr2—C20	−95.57 (12)	C30—C26—Zr3—C23	−45.70 (14)
Zr3—O3—Zr2—C16	−83.28 (13)	C27—C26—Zr3—C23	−159.89 (13)
Zr3—O3—Zr2—C19	−127.67 (13)	C30—C26—Zr3—C27	114.19 (19)
Zr3—O3—Zr2—C15	139.52 (11)	C28—C27—Zr3—O2	156.35 (13)
Zr3—O3—Zr2—C12	92.04 (12)	C26—C27—Zr3—O2	−88.99 (13)
Zr3—O3—Zr2—C13	82.39 (13)	C28—C27—Zr3—O3	58.56 (13)
Zr3—O3—Zr2—C14	115.10 (12)	C26—C27—Zr3—O3	173.22 (12)
Zr3—O3—Zr2—C17	−111.90 (12)	C28—C27—Zr3—C25	−162.8 (2)
Zr3—O3—Zr2—C11	123.42 (13)	C26—C27—Zr3—C25	−48.1 (3)
Zr3—O3—Zr2—C18	−139.77 (11)	C28—C27—Zr3—C21	−16.4 (3)
Zr1—O1—Zr2—O3	−2.42 (13)	C26—C27—Zr3—C21	98.3 (2)
Zr1—O1—Zr2—C20	81.14 (13)	C28—C27—Zr3—C24	−128.79 (13)
Zr1—O1—Zr2—C16	109.06 (13)	C26—C27—Zr3—C24	−14.12 (17)
Zr1—O1—Zr2—C19	84.70 (14)	C28—C27—Zr3—C30	−77.56 (14)
Zr1—O1—Zr2—C15	−129.00 (12)	C26—C27—Zr3—C30	37.10 (12)
Zr1—O1—Zr2—C12	−90.13 (13)	C28—C27—Zr3—C29	−36.75 (12)
Zr1—O1—Zr2—C13	−119.01 (13)	C26—C27—Zr3—C29	77.91 (14)
Zr1—O1—Zr2—C14	−142.52 (12)	C28—C27—Zr3—C22	−50.18 (17)
Zr1—O1—Zr2—C17	134.83 (12)	C26—C27—Zr3—C22	64.48 (16)
Zr1—O1—Zr2—C11	−90.07 (13)	C26—C27—Zr3—C28	114.66 (19)
Zr1—O1—Zr2—C18	125.44 (12)	C28—C27—Zr3—C23	−90.09 (14)
C16—C20—Zr2—O3	158.10 (13)	C26—C27—Zr3—C23	24.57 (16)
C19—C20—Zr2—O3	−88.25 (13)	C28—C27—Zr3—C26	−114.66 (19)
C16—C20—Zr2—O1	61.04 (13)		
